# Intravenous Fosfomycin for Gram-Negative and Gram-Positive Bacterial Infections: A Systematic Review of the Clinical Evidence

**DOI:** 10.3390/antibiotics14121193

**Published:** 2025-11-23

**Authors:** Matthew E. Falagas, Dimitrios S. Kontogiannis, Laura T. Romanos, Dimitrios Ragias, Maria Eleni Agoranou, Stylianos A. Kakoullis

**Affiliations:** 1 Alfa Institute of Biomedical Sciences (AIBS), 151 23 Athens, Greece; 2 School of Medicine, European University Cyprus, 2404 Nicosia, Cyprus; s.kakoullis@euc.ac.cy; 3 Department of Medicine, Tufts University School of Medicine, Boston, MA 02111, USA

**Keywords:** *Acinetobacter baumannii*, combination therapy, Enterobacterales, *Enterococcus*, fosfomycin, Gram-negative bacteria, Gram-positive bacteria, *Klebsiella pneumoniae*, Pseudomonas aeruginosa, *Staphylococcus aureus*

## Abstract

**Background:** The increasing worldwide problem of bacterial infections caused by multidrug-resistant Gram-negative and Gram-positive pathogens has led to consideration of intravenous fosfomycin, especially in combination antimicrobial regimens. We performed a systematic review of the evidence from comparative and non-comparative studies of patients who received intravenous fosfomycin as monotherapy or in combination with other antibiotics. **Methods:** Relevant studies were sought in six resources (Cochrane Library, Google Scholar, PubMed Central, PubMed, Scopus, and Web of Science) and two registries [ClinicalTrials.gov and International Clinical Trials Registry Portal (ICTRP)]. **Results:** Of the 2351 screened articles, 53 (31 comparative and 22 non-comparative studies) reported relevant data for patients with infections at various sites caused by Gram-negative bacteria, including Enterobacterales, *Pseudomonas aeruginosa*, and *Acinetobacter baumannii*, and Gram-positive bacteria, including *Staphylococcus* spp. and enterococci. Intravenous fosfomycin, either as monotherapy or combination therapy, showed similar or, in some studies, better efficacy (clinical and microbiological cure) compared to therapy with antimicrobial agents not including fosfomycin. The data evaluated also suggest that intravenous fosfomycin has a good safety profile. The administration of the antibiotic may be associated with electrolyte imbalances, especially hypokalemia and hypernatremia. These adverse events may be prevented and controlled with appropriate therapeutic measures and rarely lead to the discontinuation of the drug. **Conclusions:** Overall, the considerable body of published data suggests that intravenous fosfomycin is safe and effective. The antibiotic may be considered for patients with both Gram-positive and Gram-negative infections, especially in critically ill patients and/or deep-seated infections. The heterogeneity of the included studies is a limitation that prevents firm conclusions.

## 1. Introduction

Infections caused by bacteria with antimicrobial resistance are among the leading causes of death worldwide and thus pose a significant public health threat [1,2]. Multidrug-resistant (MDR) bacteria have developed several resistance mechanisms, facilitated by the improper use of antimicrobial agents and antibiotic usage in livestock [3]. While new antibiotics should be investigated for introduction into clinical practice, further research is needed on the effectiveness and safety of older antibiotics.

Fosfomycin is a broad-spectrum antibiotic that has gained renewed interest due to the potential of its intravenous formulation to combat MDR bacterial infections. It has a bactericidal action by inactivating the UDP-N-acetylglucosamine enolpyruvyltransferase (MurA) enzyme, which catalyzes the first committed step in peptidoglycan synthesis. This mechanism inhibits bacterial cell wall synthesis in an earlier stage than β-lactam antibiotics [4]. Its antimicrobial spectrum is broad, encompassing the majority of clinically relevant Gram-negative and Gram-positive bacteria, including lactose non-fermenters such as *Pseudomonas aeruginosa*. Fosfomycin is a low-molecular-weight molecule with pharmacokinetic properties that enable it to distribute well in various tissues [5].

Several in vitro studies have demonstrated synergistic effects between fosfomycin and various antimicrobial agents, such as penicillins and cephalosporins, carbapenems, chloramphenicol, aminoglycosides, sulbactam, linezolid, tetracyclines, and daptomycin [6]. These studies focused on the synergistic potential of fosfomycin against both Gram-negative, including Enterobacterales, *P. aeruginosa,* and *Acinetobacter baumannii*, and Gram-positive pathogens, including *Staphylococcus aureus* and *Enterococcus* spp. [4,7,8].

Interestingly, in addition to its pharmacokinetic and pharmacodynamic properties, some data suggest that fosfomycin may exhibit nephroprotective effects [9]. This may be particularly beneficial for patients who receive nephrotoxic drugs such as aminoglycosides [10], as well as polymyxins (colistin and polymyxin B). This is potentially due to the inhibition of iron release from mitochondria, as shown in an animal study, which led to the depression of gentamicin-induced lipid peroxidation in rat renal cortex mitochondria. It is considered to be one of the possible mechanisms that contribute to the protection of fosfomycin against gentamicin-induced nephrotoxicity [11]. Fosfomycin’s nephroprotective mechanism may also be attributed to its capacity to inhibit aminoglycoside-induced histamine release following the mast cell destruction caused by aminoglycosides [12].

In this context, a systematic review of the data on the effectiveness and safety of intravenous fosfomycin in treating patients with Gram-negative and Gram-positive infections, including comparative studies of intravenous fosfomycin combination regimens versus other antibiotics, was conducted. Such an evaluation of the published evidence may provide clinicians with insights into the clinical utility of intravenous fosfomycin in an era of rising antimicrobial resistance.

## 2. Results

### 2.1. Literature Search

**Figure 1:**
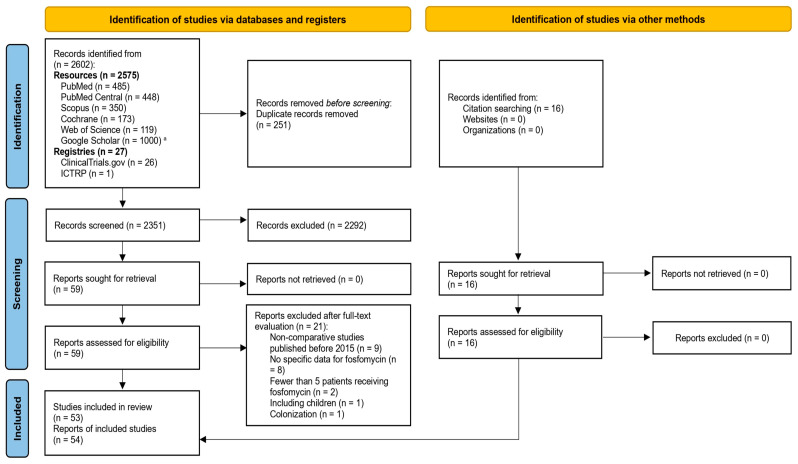
“Preferred Reporting Items for Systematic reviews and Meta-Analyses” (PRISMA) flow diagram of included studies. Notes: ^a^ For Google Scholar, out of the 7850 results, only the first 1000 articles could be accessed.

Figure 1 summarizes the results yielded by the literature search (PRISMA flow diagram). In total, 2575 records were identified across the six resources (Cochrane Library, Google Scholar, PubMed Central, PubMed, Scopus, and Web of Science), and 27 studies were identified from the two registries [ClinicalTrials.gov and International Clinical Trials Registry Portal (ICTRP)]. After deduplication, 251 studies were removed, leaving 2351 articles for evaluation based on title and/or abstract. After excluding 2292 studies, 59 were retrieved and assessed for eligibility by full text. In the final screening steps, 21 studies were excluded. Specifically, these were excluded due to publication before 2015 for the non-comparative studies (9 studies), no specific data regarding treatment with fosfomycin (8 studies), inclusion of fewer than five patients that received fosfomycin treatment (2 studies), inclusion of pediatric patients (1 study), and inclusion of patients with colonization (1 study). In addition to database searches, 16 further studies were identified through citation searching of relevant reviews. Finally, 54 reports of 53 studies were deemed eligible and included in our analysis [13,14,15,16,17,18,19,20,21,22,23,24,25,26,27,28,29,30,31,32,33,34,35,36,37,38,39,40,41,42,43,44,45,46,47,48,49,50,51,52,53,54,55,56,57,58,59,60,61,62,63,64,65].

### 2.2. Evaluation of Risk of Bias

**Figure 2:**
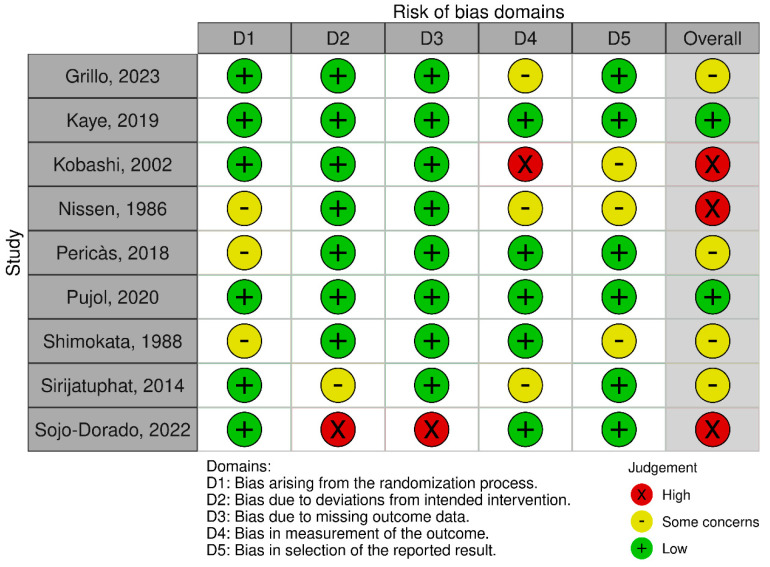
Risk of bias for randomized controlled trials (RCTs) using the “Cochrane risk-of-bias tool for randomized trials” (RoB 2) tool. Grillo, 2023 [28]; Kaye, 2019 [38]; Kobashi, 2002 [25]; Nissen, 1986 [27]; Pericàs, 2018 [32]; Pujol, 2021 [30]; Shimokata, 1988 [26]; Sirijatuphat, 2014 [24]; Sojo-Dorado, 2020 [37]”.

**Figure 3:**
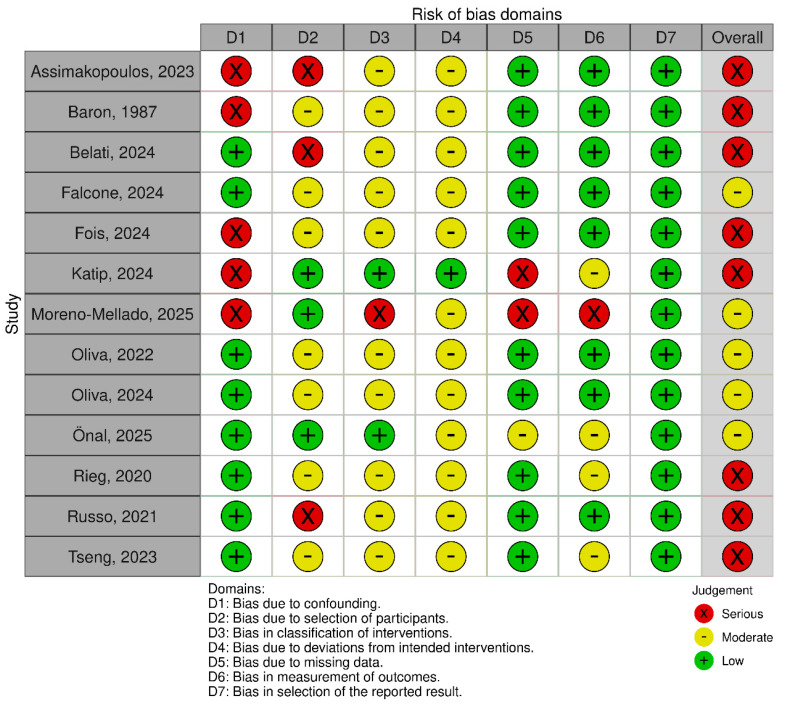
Risk of bias for observational (non-randomized) studies using the “Risk Of Bias In Non-randomized Studies–of Interventions, Version 2” (ROBINS-I V2) tool. Assimakopoulos, 2023 [18]; Baron, 1987 [30]; Belati, 2024 [14]; Falcone, 2024 [32]; Fois, 2024 [15]; Katip, 2024 [16]; Moreno-Mellado, 2025 [33]; Oliva, 2022 [19]; Oliva, 2024 [17]; Önal, 2025 [13]; Rieg, 2020 [28]; Russo, 2021 [20]; Tseng, 2023 [26].

Figure 2 and Figure 3 present the results of the risk of bias assessment for each article that included fosfomycin monotherapy or combination therapy compared with other antimicrobial regimens, using the RoB 2 and ROBINS-I V2 tools, respectively, for both Gram-negative and Gram-positive infections. The figures were created using the “risk-of-bias visualization” (robvis) tool.

### 2.3. Tabulation of Extracted Data

Table 1 presents the study characteristics, regimens received in the interventions, population characteristics, primary, and secondary outcomes of studies that included patients with Gram-negative and Gram-positive infections who received fosfomycin combination regimens compared to other antibiotic regimens [13,14,15,16,17,18,19,20,21,22,23,24,25,26,27,28,29,30]. In total, 18 studies were included. Seven studies evaluated only Gram-negative bacteria, mainly *Escherichia coli*, *Klebsiella pneumoniae*, and *A. baumannii*. Five studies included both Gram-negative and Gram-positive bacteria. Six studies included only Gram-positive bacterial pathogens, mainly *S. aureus* and *Enterococcus faecium*. Five studies originated in Italy, three in Spain, two in Japan, two in Thailand, and one each in Denmark, France, Germany, Greece, Taiwan, and Turkey. In a retrospective cohort study, the fosfomycin combination group showed statistically significantly lower 28-day all-cause mortality than combination therapy with other antimicrobial agents excluding fosfomycin in patients with PDR *A. baumannii* bloodstream infection [18]. Specifically, the 28-day mortality was 1/8 (13%) for the fosfomycin group compared to 9/12 (75%) for the other antibiotics group (*p*-value = 0.005) [18].

Table 2 presents the study characteristics, regimens received in the interventions, population characteristics, primary, and secondary outcomes of the studies that included patients with Gram-negative and Gram-positive infections who received fosfomycin monotherapy compared to other antibiotic regimens [31,32,33,34,35]. In total, four studies were included, with patients infected mainly by *E. coli*, but also by other Enterobacterales. One study was conducted in Italy, one in Spain, and one in Italy, Spain, and Turkey; another was conducted in 16 countries that were not specified.

Table 3 presents the study and population characteristics, as well as the outcomes of studies comparing fosfomycin combinations with other antibiotics to other combinations that included, among others, fosfomycin or other monotherapy regimens [31,32,36,37,38,39,40,41,42,43,44]. These studies did not provide specific data for the subset of patients receiving fosfomycin therapy. The outcomes are presented as a sum in each study group. Ten studies were included in this table. The most commonly isolated bacterial pathogen was carbapenem-resistant *A. baumannii*, but also *K. pneumoniae*. Eight studies originated in Italy, one in China, and one in Thailand. In an observational study of 73 patients in a single center who developed ventilator-associated pneumonia (VAP) and bacteremia caused by carbapenem-resistant *A. baumannii* (CRAB), the combination treatment with cefiderocol plus fosfomycin was associated with higher 30-day survival (*p*-value < 0.001) [41]. In another study, combination therapy, in which 6/41 (14.6%) patients received fosfomycin combinations, was associated with lower 30-day mortality than ceftazidime-avibactam alone (*p*-value = 0.001) [43].

Table 4 presents the study and population characteristics, the antibiotics administered, and the primary and secondary outcomes of non-comparative studies that included fosfomycin combination regimens [45,46,47,48,49,50,51,52,53,54,55,56,57,58,59,60,61,62,63]. A total of 19 studies were included. Nine evaluated only Gram-negative pathogens, most commonly *E. coli*, *A. baumannii*, *P. aeruginosa*, and *K. pneumoniae*. Seven studies evaluated both Gram-negative and Gram-positive pathogens, and three evaluated only Gram-positive pathogens. Six studies originated in Italy, two in Turkey, two in Thailand, and one each in Brazil, Canada, India, Lebanon, Qatar, Spain, and Taiwan. Additionally, one study was conducted in Germany and Austria, and another in Germany, Italy, Greece, Austria, and the United Kingdom.

Table 5 presents the study and population characteristics, the antibiotics administered, and the primary and secondary outcomes of non-comparative studies that included fosfomycin monotherapy [31,32,47,49,64,65]. Five relevant studies were included, and all evaluated only Gram-negative isolates, mainly *E. coli* and *K. pneumoniae*. One of each was conducted in Canada, Italy, Spain, Thailand, and Turkey.

## 3. Discussion

We evaluated the published evidence on the use of intravenous fosfomycin in antimicrobial combination therapy regimens or as monotherapy for the treatment of patients with infections caused by Gram-negative and Gram-positive bacteria. Our study shows that there is a considerable number of publications with data that support the effectiveness, especially in combination antimicrobial treatment, and a good safety profile of intravenous fosfomycin for patients with various types of infections, including nosocomial pneumonia (HAP/VAP), urinary tract infections (UTIs), intra-abdominal infections, bacteremia/sepsis, infective endocarditis, bone and joint infections (BJIs), and central nervous system (CNS) infections.

The evaluated data on the antimicrobial combination treatment with intravenous fosfomycin for treating patients with difficult-to-treat MDR bacterial infections deserve particular attention. Several studies have demonstrated a survival benefit in patients with infections caused by resistant Gram-negative and Gram-positive bacteria, particularly in foreign body-associated infections [28] and in more severely ill patients [14,27]. Higher 30-day survival was observed in a study of patients with VAP and associated bacteremia due to CRAB treated with the combination of cefiderocol and fosfomycin, a difference with statistical significance [41]. In another study, the 28-day mortality of patients with PDR *A. baumannii* bloodstream infection was significantly lower in the fosfomycin combination group compared to the combination of other antimicrobial agents excluding fosfomycin [18]. Additionally, in a subgroup analysis of a retrospective cohort study from Italy, a significant risk reduction was observed among patients with bloodstream infections caused by Gram-negative bacteria who had a Sequential Organ Failure Assessment (SOFA) score greater than 6 or a Pitt bacteremia score of 4 or higher [14].

In several studies, intravenous fosfomycin combination regimens showed better microbiological outcomes, especially in the early phase of treatment. Most notably, this was demonstrated in two randomized controlled trials (RCTs) that compared fosfomycin-containing regimens with monotherapy for the treatment of methicillin-resistant *S. aureus* (MRSA) or methicillin-susceptible *S. aureus* (MSSA) bacteremia [25,27,66]. For example, in the “BACSARM” trial, 0/74 (0%) patients who received fosfomycin plus daptomycin, compared with 5/81 (6.2%) patients who received daptomycin alone, had persistent bacteremia after 7 days of treatment [27]. Thus, the combination therapy of fosfomycin plus daptomycin led to a statistically significantly faster microbiological eradication [27]. Achieving a rapid microbiological cure is an important aspect when considering treatment with more than one antimicrobial agent [27].

Of note, despite superior bacterial clearance, both studies failed to demonstrate that intravenous fosfomycin combination therapy improved treatment success for their respective primary endpoints. Better microbiological outcomes were also demonstrated in an RCT that included patients with infections due to *A. baumannii* (including co-infections with other Gram-negative or Gram-positive pathogens such as MRSA), as well as a retrospective cohort study that included patients with infections caused by CRAB and *K. pneumoniae* carbapenemase (KPC)-producing *K. pneumoniae* [17,21].

In a post hoc analysis of data from a study included in our systematic review (the FOREST trial), the authors employed the desirability of outcome ranking (DOOR) methodology to evaluate further the results of their original study [34,67]. Three DOOR definitions were used, with 5, 6, or 7 categories of outcome combinations [67]. Patients receiving fosfomycin combination antimicrobial therapy had better DOOR ranking outcomes when step-down therapy with an oral drug and the ecological impact (pressure on antimicrobial resistance development) of the step-down therapy were taken into account [0.61 (0.53–0.69)] [67].

Current clinical data support the use of higher doses of intravenous fosfomycin than previously used. The dosage of fosfomycin used in the majority of the recent studies included in our analysis was 16 to 24 g per day, divided into three to four doses. This approach is supported by the fact that intravenous fosfomycin is frequently used in patients with difficult-to-treat infections and by the pharmacokinetic need to achieve therapeutic antibiotic concentrations at difficult-to-reach infection sites. Additionally, the duration of intravenous infusion varies across studies. Most studies reported 30 to 60 min infusions, with some extending to 3 h. It is still unclear whether higher or shorter duration of antimicrobial therapy is needed, especially for patients with complicated infections [68].

It is reassuring that intravenous fosfomycin, even in high daily dosages of 16 to 24 g, is not commonly associated with adverse events. However, in one study, a higher proportion of participants discontinued treatment due to adverse events when compared to the comparator study arm (17% vs. 5%) [27]. The authors suggested that the particular antibiotic combination in this study (fosfomycin and daptomycin) may be associated more frequently with electrolyte disorders, specifically hypokalemia and hypocalcemia [27]. Therefore, particular attention should be paid to the risk of hypokalemia, hypocalcemia, and hypernatremia. The formulation of fosfomycin for intravenous administration is fosfomycin disodium, which may lead to hypernatremia and fluid overload. Regular measurements of serum electrolytes, specifically sodium and potassium, and corrective therapeutic measures are necessary for the diligent management of patients treated with intravenous fosfomycin if relevant abnormalities are detected.

In this context, in the “SAFO” trial, attending physicians were advised to use supplementary potassium and low doses of diuretics to account for possible electrolyte imbalances associated with intravenous fosfomycin (and cloxacillin) [69], resulting in comparable (low) incidences of hypokalemia between the combination therapy and monotherapy arms [25]. Another measure could be a prolonged intravenous fosfomycin infusion, as a French retrospective study found that patients who received prolonged intravenous fosfomycin had significantly less hypokalemia than those who received a standard short infusion [70]. Similarly, a recently published Italian study in which intravenous fosfomycin was administered over a 3 h infusion reported no cases of hypokalemia among treated patients [17]. In most studies reporting electrolyte imbalances as adverse events of fosfomycin therapy, discontinuation of fosfomycin was not necessary. Overall, although some studies have reported an increased number of adverse events in the intravenous fosfomycin group, the safety profile of intravenous fosfomycin appears good and comparable to that of comparators, though one trial reported a significantly higher proportion of patients with adverse events leading to discontinuation of treatment in the fosfomycin combination arm, as previously mentioned [4,25,27].

Besides the increasing use of intravenous fosfomycin for the treatment of patients with MDR Gram-negative infections in many parts of the world, a development driven by clinical need, the drug is still frequently considered for the treatment of Gram-positive infections. The evaluated data in this systematic review indicate that intravenous fosfomycin has been used in many countries to treat staphylococcal and enterococcal infections at various body sites, including bacteremia (including catheter or central line-associated bloodstream infections), endocarditis, intra-abdominal infections, and prosthetic joint infections [55]. The published evidence suggests that intravenous fosfomycin may be beneficial as an adjunctive treatment for patients with Gram-positive bacterial infections. For example, a pooled post hoc analysis of data from the two aforementioned multicenter RCTs, specifically the “BACSARM” and “SAFO” trials, showed that the addition of intravenous fosfomycin to daptomycin or cloxacillin for MRSA and MSSA bacteremia was associated with statistically significant improvement in early bacterial clearance and treatment success at 8 weeks. Both “BACSARM” and “SAFO” trials were terminated prematurely before reaching their planned sample sizes. Thus, they had limited statistical power [66]. Additionally, the trials included a large proportion of patients with catheter-related and noncomplicated bacteremia [66]. These infections have a more favorable prognosis and may have masked the potential benefits of the addition of fosfomycin for patients with more severe disease [66]. Additionally, the higher incidence of adverse events, which led to treatment discontinuation, possibly counteracted the advantage of accelerating bacterial clearance [66].

A few previous reviews have examined the published data on the effectiveness of intravenous fosfomycin, primarily in combination with other antimicrobial agents, for treating patients with difficult-to-treat infections, including BJIs and CNS infections [71,72,73,74,75]. This highlights the consideration of combination antimicrobial agents, including intravenous fosfomycin, for infections caused by highly resistant isolates and difficult-to-reach infection sites, particularly when other antibiotic treatments have been unsuccessful. Our analysis supports the available data from other published reviews addressing the treatment of patients with difficult-to-treat Gram-negative and Gram-positive bacterial infections. Real-world data indicate that intravenous fosfomycin is primarily used in combination therapy as empirical treatment, as targeted therapy (either first- or second-line), or as salvage therapy [55,58].

Intravenous fosfomycin is primarily used as an adjunct to the antibiotic backbone in patients with difficult-to-treat infections. However, available data indicate that it may also be a promising carbapenem-sparing agent for monotherapy of UTIs caused by extended-spectrum β-lactamase (ESBL)-producing Enterobacterales [33,34,35,67].

As mentioned earlier, several in vitro studies have demonstrated that fosfomycin exhibits considerable synergistic activity with several antimicrobial agents [7,76,77,78], which is one of the main rationales for its primary use as an adjunct to the antibiotic backbone. Additionally, fosfomycin has pharmacokinetic and pharmacokinetic/pharmacodynamic (PK/PD) properties of clinical significance, including penetration into various sites of infection, such as the respiratory system or cerebrospinal fluid [79,80,81]. Moreover, the antibiotic exhibits antimicrobial activity against microbial biofilms [74]. This characteristic is significant for various types of infections, especially BJIs such as prosthetic joint infections, and may explain its use in these types of infections. Furthermore, fosfomycin has demonstrated antimicrobial activity against a significant proportion of bacteria with advanced antimicrobial resistance profiles [82], rendering it a valuable option for empirical therapy in regions with high resistance rates. While several mechanisms of fosfomycin resistance development in bacterial pathogens have been described, available data suggest that such resistance is uncommon [83,84,85,86].

Our study has several strengths that distinguish it from previous reviews of the clinical evidence for intravenous fosfomycin. Firstly, the majority of the articles included in our systematic review are recent relevant publications that have not yet been summarized in previous reviews. Secondly, both Gram-negative and Gram-positive infections are included in our analysis, regardless of the specific isolates involved or the infection site. Thirdly, our systematic review compares fosfomycin combination regimens with other antibiotic combinations or monotherapy that do not include fosfomycin; thus, the effectiveness of the intervention was assessed using a comparator group. However, a limitation of this study is the high heterogeneity among the evaluated studies, including varying degrees of infection severity (e.g., inclusion of critically ill patients), different types of infections, and varied dosing schemes. Subgroup and sensitivity analyses based on various parameters, including infection site, pathogen, patient comorbidity, or fosfomycin dosage schemes, as well as syndrome-specific summary figures, would provide useful information for the clinicians. However, the limited number of studies for groups of patients with similar characteristics due to study heterogeneity does not permit such analyses. Additionally, a detailed analysis of electrolyte imbalances was not possible due to the limited data available in the included studies. Another limitation is that most data come from observational studies rather than RCTs, which limits the conclusions that can be drawn. A meta-analysis was not undertaken, nor was a certainty-of-evidence analysis conducted, because of the considerable heterogeneity of the studies included in our article. Lastly, a limitation of this study is that it was not registered in a registry platform, such as PROSPERO.

## 4. Methods

### 4.1. Adherence to the PRISMA Guidelines

The methods of our article comply with the most recent “Preferred Reporting Items for Systematic Reviews and Meta-Analyses” (PRISMA) guidelines. Any omission is reported in the Section 3 of this article. The study research protocol was not registered in a database. The PRISMA checklists for both the abstract and full-text article are provided in Supplementary Files S1 and S2, respectively.

### 4.2. Eligibility Criteria

Studies were eligible for evaluation if they included adult patients with infections (systemic or localized) requiring antibiotic treatment, regardless of the site of infection and the presence of foreign bodies or implanted devices. The causative pathogens included both Gram-negative and Gram-positive bacteria. Regarding the intervention, studies were eligible for inclusion in our article if patients received intravenous fosfomycin, either as monotherapy or in combination with other antibiotic(s), with or without a comparison group.

Studies with patients who received combination antimicrobial therapy [with a subset receiving fosfomycin with other antimicrobial(s)] and a comparison group that received either monotherapy or combination therapy were also included. All antibiotic agents used should have been administered intravenously. There were no limitations on dosages and treatment duration. The primary outcome was all-cause mortality. Secondary outcomes were clinical cure or a similar endpoint (as reported by the treating physicians), microbiological cure, and adverse events related to treatment.

Studies with fewer than five patients receiving fosfomycin were excluded from further evaluation, as the data from these studies would be limited for interpretation. Furthermore, studies that did not report any of the outcomes (primary and/or secondary) were excluded. For the non-comparative studies, we included studies published from 2015 to the time of the implemented search strategies, as previous studies had summarized relevant data prior to 2015 [71].

### 4.3. Search Strategy

A systematic search was conducted to identify relevant studies across six resources (PubMed, PubMed Central, Scopus, Cochrane Central Registry of Controlled Trials, Web of Science, and Google Scholar) and two registries (ClinicalTrials.gov and the International Clinical Trials Registry Portal). The above databases and registries were searched from inception to 12 August 2025, for PubMed Central, and to 6 August 2025, for the remaining resources. No additional filters were applied regarding the language or year of publication. For Google Scholar, only the first 1000 results could be accessed. The search string strategies are presented in Supplementary File S3.

To enhance sensitivity, only terms related to the population under study (excluding pediatric patients) and the type of intervention (intravenous fosfomycin antibiotic regimens, either combination therapy or monotherapy) were used. In addition, reference lists of relevant articles were searched for otherwise non-identified reports.

### 4.4. Screening Process and Data Extraction

Two reviewers (D.S.K. and L.T.R.) independently completed the screening process in a semi-automated manner, utilizing the “Rayyan” software (https://www.rayyan.ai/, accessed on 5 August 2025). Screening was performed first by title and/or abstract, and then by full text. An exception was PubMed Central, where the studies were screened by full text. Discrepancies were resolved by consensus, with a third reviewer (D.R.) and, if needed, by a senior author (M.E.F.). Data extraction and tabulation were performed by two authors (D.S.K. and L.T.R.), who also validated their accuracy. When more than one publication reported on the same patient cohort, data from the most complete publication were used in our analysis.

### 4.5. Risk of Bias Assessment

The risk of bias was assessed using the “Cochrane risk-of-bias tool for randomized trials” (RoB 2) [87] and the “Risk Of Bias In Non-randomized Studies–of Interventions, Version 2” (ROBINS-I V2) tools for randomized studies and non-randomized cohort studies, respectively [88]. Studies including a fosfomycin combination group or monotherapy in comparison to other antimicrobial agent(s) were assessed for their risk of bias. Two authors (D.S.K. and L.T.R.) independently evaluated the risk of bias, and any discrepancies were resolved by consensus with a senior author (M.E.F.). Studies were classified, according to the respective algorithms of each tool, as “low risk of bias, some concerns, or high risk of bias” using the RoB 2 tool and as “low, moderate, serious, or critical risk of bias” using the ROBINS-I V2 tool. Studies classified as “critical risk of bias”, if any, were excluded from the systematic review.

## 5. Critical Appraisal of the Available Evidence

A considerable number of articles from various countries reported on the effectiveness and safety of intravenous fosfomycin for treating patients with Gram-negative and Gram-positive infections. Our systematic review of the relevant published data supports consideration of intravenous fosfomycin for both Gram-negative and Gram-positive bacterial infections, including those caused by MDR or extensively drug-resistant (XDR) bacteria.

Due to its favorable pharmacokinetics and broad spectrum of activity, intravenous fosfomycin is suitable for both empirical and targeted therapy, as well as salvage therapy. Current clinical practice guidelines and expert opinions support these applications and highlight the place of intravenous fosfomycin in the antibiotic landscape [89,90,91,92].

The published data indicate that critically ill patients, as well as patients with difficult-to-treat infections or infections caused by highly resistant pathogens, could particularly benefit from the addition of intravenous fosfomycin to the antibiotic regimen [14,17,27,46]. Additionally, fosfomycin could be valuable for patients with foreign body infections, deep-seated infections, and CNS infections (a difficult-to-reach compartment) [28,55] or in infections where time to microbiological eradication is crucial. For example, in *S. aureus* bacteremia, the duration to eradication appears to be associated with mortality [93,94]. In this regard, various studies have shown faster microbiological eradication or fewer microbiological failures in fosfomycin combination regimens [17,21,25,26,27,66]. Interestingly, early initiation of intravenous fosfomycin treatment appears to be beneficial for clinical outcomes [14], reinforcing the idea to “hit hard and early” with intravenous fosfomycin as an adjunct to the antibiotic backbone.

Based on available clinical data, intravenous fosfomycin may also serve as an alternative to the standard of care (SOC) with carbapenems for patients with complicated UTIs caused by ESBL-producing Enterobacterales, especially in regions with higher proportions of carbapenem resistance. Interestingly, intravenous fosfomycin appears to be even effective as monotherapy against MBL-producing Enterobacterales and KPC-producing *K. pneumoniae* [31,64]. However, data are currently limited and warrant further clinical studies to confirm these results.

Although fosfomycin on its own exhibits only low in vitro susceptibility against *A. baumannii* [18,41], data from the included studies in our analysis suggest that intravenous fosfomycin may also have a role as an adjunct to the antibiotic backbone, especially in combination with cefiderocol, for patients with severe infections caused by *A. baumannii* [18,41].

As noted earlier, most published data originate from observational studies (prospective and retrospective), which carry inherent limitations in the conclusions that can be drawn. However, real-world evidence deserves particular attention as a complement to RCTs, as controlled trials may exhibit limitations regarding generalizability. For example, a systematic review showed that mortality in patients with *S. aureus* bacteremia was consistently lower in RCTs than in observational studies, due to the stricter eligibility criteria [95]. In this context, other studies have also shown differences in patient characteristics between RCTs and observational studies [96,97,98]. While RCTs remain the gold standard, they are often time-consuming and more costly than observational studies, and the highly selected patient populations may not resemble patients treated in daily clinical practice [97]. Thus, although well-designed RCTs are needed to confirm the added value of intravenous fosfomycin-containing regimens over monotherapy or SOC, real-world data provide valuable insights into the effectiveness of intravenous fosfomycin across diverse patient populations.

More generally, the potential benefit of combination therapy vs. monotherapy remains a subject of ongoing debate and research, and study design considerations may explain specific findings. For example, the absence of positive results for the primary endpoint in *S. aureus* bacteremia trials comparing fosfomycin combination therapy to monotherapy may be attributed to the considerable number of catheter-related bacteremia cases and the inclusion of mostly moderately ill patients [25,26,27]. However, as discussed above, intravenous fosfomycin combination therapy may be particularly beneficial in more severely ill patients or in cases of bacteremia involving foreign bodies/deep foci [27,55]. In this context, the ongoing innovative “SNAP” trial (*S. aureus* bacteremia Network Adaptive Platform Trial) is planning to include a fosfomycin domain for patients with *S. aureus* bloodstream infections. It may reveal whether intravenous fosfomycin provides added value as an adjunctive therapy in treating (complicated) *S. aureus* bacteremia [99].

The continuing interest in intravenous fosfomycin is evidenced by several ongoing clinical trials, notably the randomized controlled “CAVIFOS” trial (NCT07063095) evaluating ceftazidime (with or without avibactam) combined with intravenous fosfomycin versus ceftazidime (with or without avibactam) monotherapy in the treatment of severe Gram-negative infections, along with other studies [for example the “TREAT-GNB” (NCT07004049) trial, trials for CRAB infections (NCT06440304, NCT06570850), for real-world use (NCT06814899), the “NeoSep1” (ISRCTN48721236), and the “PROOF” (NCT05211011) trials] [100,101,102,103,104,105,106].

## 6. Conclusions

In conclusion, intravenous fosfomycin has demonstrated effectiveness and a favorable safety profile for treating patients with various infections caused by both Gram-negative and Gram-positive bacteria, including MDR and/or XDR pathogens, particularly when used in combination regimens for critically ill patients with difficult-to-treat infections. Additionally, the available data suggest that intravenous fosfomycin may also be considered as a carbapenem-sparing agent for monotherapy of patients with UTIs caused by ESBL-producing Enterobacterales.

## Figures and Tables

**Table 1:** Characteristics and outcomes of patients treated with intravenous fosfomycin combination regimens vs. other antibiotic(s).

Author, Year	Type of Study	Fosfomycin N	Companion to Fosfomycin *n*	Comparator N	Comparator *n*	Population Characteristics Total or Fosfomycin Group vs. Comparator Group Mean ± SD or Median (IQR) *n*/N (%)	Infection Type(s) Total or Fosfomycin Group vs. Comparator Group *n*/N (%)	Pathogen(s) Total or Fosfomycin Group vs. Comparator Group *n*/N (%)	Fosfomycin Dosage g/d	Mortality Fosfomycin Group vs. Comparator Group *n*/N (%)	Clinical Cure Fosfomycin Group vs. Comparator Group *n*/N (%)	Microbiological Cure Fosfomycin Group vs. Comparator Group *n*/N (%)	Adverse Events Fosfomycin Group vs. Comparator Group *n*/N (%)
Belati, 2024 [14]	Retrospective cohort	98	98 (targeted therapy) 46 carbapenem or carbapenem/BLIs 35 cephalosporin or cephalosporin/BLIs 14 other BL/BLIs 3 other drugs 7/98 additional fluoroquinolones or combination with >2 drugs	265	265 (targeted therapy) 100 carbapenem or carbapenem/BLIs 100 cephalosporin or cephalosporin/BLIs 56 other BL/BLIs 9 other drugs 174/265 monotherapy 91/265 combination 46 aminoglycosides 16 tigecycline 2 colistin 26 fluoroquinolones or combinations with >2 drugs	Age 68 (IQR 57–78) y; males 211/363 (58); ward: medical 223/363 (61), surgical 86/363 (24), ICU 54/363 (15); Pitt score ≥ 4 points 115/363 (32); comorbidities: COPD 28/98 (29) vs. 44/265 (17), *p* = 0.01; obesity 14/98 (14) vs. 17/265 (6), *p* = 0.02; acute kidney failure 41/98 (42) vs. 81/265 (31), *p* = 0.04; deep site-associated bloodstream infection 40/98 (41) vs. 74/265 (28), *p* = 0.02	BSI 363/363 (100)	*K. pneumoniae* 151/363 (42)*, E. coli* 102/363 (28), *P. aeruginosa* 63/363 (17)*, S. maltophilia/B. cepacia/Achromobacter xylosoxidans* 42/363 (12), *E. cloacae complex/K. aerogenes/C. freundii* 38/363 (10)	Median (IQR): 16 (16–18)	All-cause 14-day 9/98 (9) vs. 53/265 (20), *p* = 0.02; all-cause 30-day 19/98 (19) vs. 71/265 (27), *p* = 0.15; protective effect of fosfomycin combinations [multivariate analysis: aHR (95% CI) 0.51 (0.28–0.92), *p* = 0.03; IPTW-adjusted multivariable analysis: aHR (95% CI) 0.53 (0.31–0.91), *p* = 0.02]	80/98 (82) vs. 188/265 (71)	63/70 (90) vs. 147/186 (79), *p* = 0.04	12/98 (12) vs. 11/265 (5), *p* = 0.02 ^b^
Fois, 2024 [15]	Retrospective cohort	41	Ceftazidime/avibactam	34	Ceftazidime/avibactam	Age 65 (IQR 57–73) y; males 61/75 (81); ICU 47/75 (63); CCI 4 (IQR 2–6)	HAP/VAP 75/75 (100)	*K. pneumoniae* 31/63 (49), *P. aeruginosa* 28/63 (44), 1/63 (2) of each: *E. coli*, *K. aerogenes*, *K. pneumoniae/P. aeruginosa*, *K. pneumoniae/P. aeruginosa/E. coli*; 43/63 (68) carbapenem-resistant; 63 patients had isolates detected	NR	28-day 11/41 (27) vs. 8/34 (24); unadjusted Cox-regression analysis for 28-day: HR (95% CI) 1.14 (0.46–2.83), *p* = 0.78; adjusted Cox-regression analysis for 28-day: HR (95% CI) 0.32 (0.07–1.39), *p* = 0.13	NR	NR	Urticaria 1 vs. 0, AKI 0 vs. 1, multiorgan failure 0 vs. 1
Katip, 2024 [16]	Retrospective cohort	153	Colistin	67	Colistin	Males 90/153 (59) vs. 41/67 (61); APACHE II 9.9 ± 6.7 vs. 8.2 ± 6.4	UTI 79/153 (52) vs. 38/67 (57), pneumonia 46/153 (30) vs. 13/67 (19), bacteremia 12/153 (8) vs. 4/67 (6), others 15/153 (10) vs. 13/67 (19)	*E. coli* 57/153 (37) vs. 26/67 (39), *K. pneumoniae* 57/153 (37) vs. 22/67 (33), *E. cloacae* 39/153 (25) vs. 19/67 (28)	8 (divided into 2 daily doses)	30-day 40/153 (26) vs. 12/67 (18), *p* = 0.19; EOT 42/153 (28) vs. 13/67 (19), *p* = 0.21	110/153 (72) vs. 57/67 (85), *p* = 0.04; propensity score analysis: aOR (95% CI) 1.48 (0.61–3.59), *p* = 0.38	147/153 (96) vs. 62/67 (93), *p* = 0.27; propensity score analysis: aOR (95% CI) 0.66 (0.18–2.38), *p* = 0.53	NR
Oliva, 2024 [17]	Retrospective cohort	37	20 ceftazidime/avibactam, 7 cefiderocol 3 meropenem, 3 meropenem/vaborbactam, 1 of each: cefiderocol + tigecycline, cefiderocol + ampicillin/sulbactam, colistin, or colistin + ampicillin/sulbactam	41	15 cefiderocol + ampicillin/sulbactam, 7 ceftazidime/avibactam + meropenem, 7 colistin + ampicillin/sulbactam, 7 colistin + meropenem 2 cefiderocol + tigecycline, 1 of each: ceftazidime/avibactam + colistin, colistin + tigecycline, colistin + ampicillin/sulbactam + tigecycline	Age 67 (IQR 53–74) y; males 33/78 (33); CCI 4 (IQR 2–5), SAPS II 33 (IQR 26–40)	VAP 30/78 (39), BSI 20/78 (26), CLABSI 15/78 (19), CNS 5/78 (6), HAP 5/78 (6), UTI 1/78 (1), SSTI 1/78 (1), IAI 1/78 (1)	CRAB 44/78 (56), KPC-producing *K. pneumoniae* 34/78 (44)	Median (IQR): 24 (16–24) (divided into 3–4 doses; 3 h infusion)	7-day mortality 0/37 (0) vs. 6/41 (15), *p* = 0.03; 14-day mortality 1/37 (3) vs. 9/41 (22), *p* = 0.02; 30-day mortality 5/37 (14) vs. 14/41 (35), *p* = 0.04 ^c^	33/37 (89) vs. 27/41 (66), *p* = 0.02; early clinical improvement 29/37 (78) vs. 21/41 (51), *p* = 0.02	28/32 (88) vs. 23/37 (62), *p* = 0.03	AKI 3/37 (8) vs. 5/41 (12), *p* = 0.7; transient increase in transaminases 1/37 (3) vs. 0/41 (0)
Assimakopoulos, 2023 [18]	Retrospective cohort	8	1 of each: colistin + co-trimoxazole, colistin, colistin + ampicillin/sulbactam + amikacin, colistin + tigecycline, tigecycline + ampicillin/sulbactam + amikacin, colistin + tigecycline, colistin + meropenem + co-trimoxazole + gentamicin, colistin + tigecycline + amikacin + co-trimoxazole	12	2 colistin + tigecycline, 1 of each: colistin + meropenem, colistin + tigecycline + ampicillin/sulbactam + co-trimoxazole + amikacin, colistin + meropenem + tigecycline + ampicillin/sulbactam, colistin + amikacin, colistin + piperacillin/tazobactam, meropenem + gentamicin, colistin + meropenem, meropenem + ampicillin/sulbactam + tigecycline, colistin + tigecycline + ampicillin/sulbactam, colistin + meropenem	Age 62 ± 14 y; males 9/20 (45); ICU 20/20 (100)	BSI 20/20 (100)	PDR-*A. baumannii* 20/20 (100)	24 (divided in 3 daily doses; 3 h infusion)	All-cause 28-day 1/8 (13) vs. 9/12 (75), *p* = 0.005	NR	8/8 (100) vs. 6/12 (50)	NR
Oliva, 2022 [19]	Retrospective cohort	61	Ceftazidime/avibactam	61	40 meropenem + ceftazidime/avibactam, 8 ceftazidime/avibactam + gentamicin, tigecycline or colistin ^d^	Age 68 (IQR 57–78) y; males 84/122 (69); hospitalized 122/122 (100); CCI 6 (IQR 5–9), Pitt score 2 (1–4)	UTI 36/122 (30), BSI 34/122 (28), LRTI 22/122 (18) [VAP 14/22 (64)], IAI 26/122 (21), CLABSI 4/122 (3)	KPC-*K. pneumoniae* 122/122 (100)	Median (IQR): 16 (12–24)	7-day 3/61 (5) vs. 3/61 (5), *p* = 1.0; 14-day 6/61 (10) vs. 5/61 (8), *p* = 0.75; 30-day 9/61 (15) vs. 11/61 (18), *p* = 0.81; higher mortality in ceftazidime/avibactam group compared to fosfomycin + ceftazidime/avibactam group, *p* = 0.05	46/61 (75) vs. 37/61 (61), *p* = 0.12	72 h 33/43 (77) vs. 35/37 (95), *p* = 0.03; 7-day 30/34 (88) vs. 28/29 (97), *p* = 0.22; 14-day 25/26 (96) vs. 17/19 (90), *p* = 0.38 ^e^	Secondary infection 17/61 (28) vs. 25/61 (41), *p* = 0.18; death associated with secondary infection 1/61 (2) vs. 7/61 (12), *p* = 0.02
Russo, 2021 [20]	Prospective cohort	44	11 colistin, 8 carbapenem + tigecycline, 7 rifampicin, 7 colistin + tigecycline, 6 tigecycline, 3 carbapenem, 2 aminoglycosides	136	Various combinations with colistin, tigecycline, aminoglycoside, rifampicin, ampicillin/sulbactam, cotrimoxazole, vancomycin, or carbapenem	Age 66 ± 16 vs. 64 ± 16 y; males 122/180 (68); ward: ICU (79), medical (19), surgical (2); CCI 6.3 ± 1.6 vs. 5.6 ± 1.8, SAPS II 43.9 ± 13.2 vs. 44.1 ± 15.3; comorbidities COPD 27/44 (61) vs. 49/136 (36), *p* = 0.005; previous MDR infections during hospital stay 4/44 (9) vs. 50/136 (37), *p* < 0.001	HAP 180/180 (100)	MDR-*A. baumannii* 180/180 (100) [out of 112 strains assessed: 31% resistant to fosfomycin; 98% XDR; 2% PDR]	12–24 (divided into 3–4 daily doses)	30-day 7/44 (16) vs. 94/136 (69), *p* < 0.001; fosfomycin combinations associated with 30-day survival: HR (95% CI) 0.04 (0.01–0.13), *p* < 0.001	NR	NR	Cardiovascular events after infection onset 14/44 (32) vs. 55/136 (40), *p* = 0.37
Önal, 2025 [13]	Retrospective cohort	41	Meropenem-based regimens; 29 combined treatment approach [17 polymyxin B/colistin, 12 aminoglycosides (amikacin or gentamicin), other]	30	Ceftazidime/avibactam-based regimens; 19 combined treatment approach [10 polymyxin B/colistin, 10 aminoglycosides (amikacin or gentamicin), 1 meropenem, 3 tigecycline, 5 cotrimoxazole]	Age 59 ± 3 y; males 44/71 (62)	BSI 24/41 (59) vs. 17/30 (57)	*Acinetobacter* spp. 11/41 (27) vs. 9/30 (30), *P. aeruginosa* 4/41 (10) vs. 5/30 (17), *Staphylococci* 6/41 (15) vs. 3/30 (10), other 2/41 (5) vs. 4/30 (13) ^a^	12–16 (divided into 2–4 doses; individual doses of a maximum of 8 g)	14-day 17/41 (42) vs. 10/30 (33), *p* = 0.49; 30-day 25/41 (61) vs. 15/30 (50), *p* = 0.36	NR	NR	NR
Sirijatuphat, 2014 [21]	RCT	47	Colistin + carbapenem 4/47 (9), piperacillin/tazobactam 1/47 (2), vancomycin 1/47 (2), or other (levofloxacin, metronidazole, amphotericin B) 2/47 (4)	47	Colistin + carbapenem 8/47 (17), piperacillin/tazobactam 1/47 (2), vancomycin 2/47 (4), or other (levofloxacin, metronidazole, amphotericin B) 3/47 (6)	Age 67 ± 17 vs. 69 ± 16 y; males 20/47 (43) vs. 24/47 (51); APACHE II 23.0 ± 6.4 vs. 21.9 ± 7.9	Pneumonia 37/47 (79) vs. 35/47 (75), primary BSI 2/47 (4) vs. 3/47 (6), UTI 3/47 (6) vs. 2/47 (4), SSTI 2/47 (4) vs. 1/47 (2), IAI/GI 2/47 (4) vs. 4/47 (9), CNS 0/47 (0) vs. 1/47 (2), others 1/47 (2) vs. 1/47 (2)	CRAB 94/94 (100); coinfection with: *P. aeruginosa* 3/47 (6), *K. pneumoniae* 2/47 (4), MRSA 2/47 (4), other 1/47 (2) vs. *K. pneumoniae* 6/47 (13), MRSA 2/47 (4), *P. aeruginosa* 1/47 (2), other 1/47 (2)	8	All-cause 28-day (47) vs. (57), *p* = 0.41; infection-related 28-day (21) vs. (28), *p* = 0.63	After 72 h: (71) vs. (66), *p* = 0.66; EOT (60) vs. (55), *p* = 0.84	72 h (91) vs. (58), *p* = 0.001; EOT (100) vs. (81), *p* = 0.01	AKI (53) vs. (60), *p* = 0.68; abnormal liver function test (13) vs. (13), *p* = 1.0
Kobashi, 2002 [22]	RCT	18	Sulbactam-cefoperazole	17	Sulbactam-cefoperazole	Age 73 (range 32–92) vs. 69 (range 36–86) y; males 15/18 (83) vs. 12/17 (71)	Pneumonia 35/35 (100)	2/18 (11) of each: *S. pneumoniae*, MSSA, MRSA, *P. aeruginosa*, 1/18 (6) of each: *K. pneumoniae*, *K. oxytoca*, *S. milleri*, *A. baumannii* vs. *K. pneumoniae* 3/17 (18), *S. pneumoniae* 2/17 (12), MSSA 2/17 (12), 1/17 (6) of each: *P. aeruginosa*, *H. influenzae*, *E. cloacae*, *K. oxytoca*, *S. marcescens*	4 (divided into 2 daily doses; 30 min infusion)	NR	17/18 (94) vs. 15/17 (88)	5/10 (50) vs. 5/8 (63)	1/18 (6) vs. 0/17 (0); severe gastrointestinal symptoms, leading to discontinuation of treatment
Shimokata, 1988 [23]	RCT	41	Cefotaxime	32	Cefotaxime	Males 29/41 (71) vs. 22/32 (69)	Pneumonia 29/41 (71) vs. 25/32 (78), secondary infection of chronic respiratory distress 6/41 (15) vs. 4/32 (13), bronchitis 3/41 (7) vs. 1/32 (3), pleurisy 0/41 (0) vs. 1/32 (3), pyothorax 0/41 (0) vs. 1/32 (3), suppurative lung disease 3/41 (7) vs. 0/32 (0)	*S. agalactiae* 2/41 (5), *H. influenzae* 2/41 (5), *K. pneumoniae* 2/41 (5), 1/41 (2) of each: *S. aureus*, *S. viridans*, *Klebsiella* spp., *P. aeruginosa*, *S. aureus* + *S. viridans*, *S. epidermidis* + *H. parainfluenza*, *H. influenzae* + *K. pneumoniae* vs. 1/32 (3) of each: *S. agalactiae*, *S. viridans*, *S. pneumoniae*, *E. aerogenes*, *P. aeruginosa*, *H. influenzae* + *K. pneumoniae*, *Acinetobacter* spp. + *Pseudomonas* spp.	2–4	NR	Total improvement 31/41 (76) vs. 26/32 (81); in severe disease 7/9 (78) vs. 1/2 (50), in moderate disease 17/25 (68) vs. 17/22 (77), in mild disease 7/7 (100) vs. 8/8 (100)	Total improvement 31/41 (76) vs. 26/32 (81); in severe disease 7/9 (78) vs. 1/2 (50), in moderate disease 17/25 (68) vs. 17/22 (77), in mild disease 7/7 (100) vs. 8/8 (100)	5/41 (12) (3 elevated AST/ALT, 1 fever, 1 vascular pain, 1 elevated BUN) vs. 4/32 (13) (1 fever, 1 thrombocytopenia, 1 elevated AST/ALT, 1 elevated ALT)
Nissen, 1986 [24]	Randomized trial	17	Ampicillin	15	Ampicillin + gentamicin	Age 57 ± 19 vs. 58 ± 21 y; males 9/17 (53) vs. 9/15 (60)	Pneumonia 3/17 (18), chronic bronchitis with respiratory insufficiency 3/17 (18), cardiovascular resuscitation after acute myocardial infarction with cardiac arrest 1/17 (6), post-operative pneumonia 1/17 (6), respiratory insufficiency 1/17 (6) vs. cardiovascular resuscitation after acute myocardial infarction with cardiac arrest 2/15 (13), pneumonia 3/15 (20), chronic bronchitis with respiratory insufficiency 2/15 (13), post-operative pneumonia 1/15 (7)	*E. coIi* 5/17 (29), coagulase-positive staphylococci 3/17 (18), 2/17 (12) of each: pneumococci, α-hemolytic streptococci, *B. catarrhalis*, *K. pneumoniae*, *P. aeruginosa*, 1/17 (6) of each: β-hemolytic streptococcus Group A, *H. influenzae, E. cloacae* vs. coagulase-positive *staphylococci* 6/15 (40), *E. coIi* 5/15 (33), *Pneumococcus* 4/15 (27), α-hemolytic streptococci 2/15 (13), *B. catarrhalis* 2/15 (13), 1/15 (7) of each: coagulase-negative *staphylococci*, β-hemolytic streptococcus Group C, *H. influenzae*, *P. aeruginosa*	12 (divided into 3 daily doses; 30 min infusion)	NR	10/17 (59) vs. 7/15 (47)	18/21 (88) vs. 18/20 (90)	Phlebitis 2/3 (67) (out of 3 who received fosfomycin from a peripheral vein), mild transient elevation in AST 1, pain 1 (that disappeared with a slower administration rate) vs. none
Grillo, 2023 [25]	Randomized clinical trial (phase III-IV)	101	Cloxacillin	106	Cloxacillin	Age 64 (IQR 55–72) vs. 68 (IQR 54–77) y; males 69 (66) vs. 81 (74); CCI 4.0 ± 3.1 vs. 4.7 ± 3.5, Pitt score 0.3 ± 0.6 vs. 0.3 ± 0.6	BSI 207/207 (100)	MSSA 207/207 (100)	12 (divided into 4 daily doses)	All-cause 7-day 2/101 (2) vs. 0 (0), *P* = 0.15; all-cause at EOT 10/101 (10) vs. 11/106 (10), *p* = 0.91; all-cause at TOC 10/101 (10) vs. 14/106 (13), *p* = 0.46	7-day 81/101 (80) vs. 81/106 (76), *p* = 0.51	7-day 81/101 (80) vs. 81/106 (76), *p* = 0.51; persistent bacteremia at day 3 4/94 (4) vs. 17/99 (17), *p* = 0.005	42/104 (40) vs. 48/110 (44), *p* = 0.73; hypokalemia 18 (17) vs. 11 (10), *p* = 0.17;
Tseng, 2023 [26]	Retrospective cohort	48	Daptomycin	176	Daptomycin	Age 67 (IQR 59–78) y; males 132/224 (58.9); CCI 3 (IQR 2–5.5) vs. 4 (IQR 2–5.5), Pitt score 1 (IQR 0–3) vs. 2 (IQR 1–4)	BSI 224/224 (100)	Vancomycin-resistant *E. faecium* 224/224 (100)	12 (6–21)	In-hospital 23/48 (48) vs. 136/176 (77), *p* < 0.001; 14-day 18/48 (38) vs. 89/176 (51), *p* = 0.11; 28-day 21/48 (44) vs. 109/176 (62), *p* = 0.02	26/48 (54) vs. 64/176 (36)	25/33 (76) vs. 60/120 (50) ^f^	Elevated CK 2/48 (4) vs. 19/176 (11), thrombocytopenia 6/48 (17) vs. 29/176 (28), hypernatremia 5/48 (10) vs. 5/176 (3), hypokalemia 16/48 (33) vs. 27/176 (15), salt overload with clinical manifestations 3/48 (6) vs. 6/176 (3), AKI 6/48 (13) vs. 41/176 (23), discontinuation due to ADE 0/48 (0) vs. 1/176 (1)
Pujol, 2021 [27]	Randomized clinical trial (phase III)	74	Daptomycin	81	Daptomycin	Age 74 (IQR 61–81) vs. 72 (IQR 62–80) y; males 104/155 (67); CCI 3 (IQR 2–5) vs. 4 (IQR 2–5.8), Pitt score 1.15 ± 1.7 vs. 1.22 ± 2.0	BSI 155/155 (100), left-sided endocarditis 18/155 (12); recurrent bacteremia 0/74 (0) vs. 4/81 (4), complicated bacteremia 12/74 (16) vs. 26/81 (32), *p* = 0.02	MRSA 155/155 (100)	12 (60 min infusion)	Overall at day-7 3/74 (4) vs. 6/81 (7); overall at TOC 18/74 (24) vs. 22/81 (27)	6 weeks after EOT 40/74 (54) vs. 34/81 (42); RR (95% CI) 1.29 (0.93–1.8)	74/74 (100) vs. 72/81 (89)	Leading to treatment discontinuation 13/77 (17) vs. 4/83 (5), *p* = 0.013 ^g^
Rieg, 2020 [28]	Prospective cohort	58	Combination therapy (not specified)	242	Rifampicin	Median age 67 y; patients with implanted foreign devices, native valve endocarditis, or osteoarticular infections; CCI 3 (IQR 1–5) vs. 3 (IQR 2–5)	BSI 300/300 (100)	MSSA 514/1156 (44), MRSA 64/1156 (6); among all studied patients	15	90-day implanted foreign devices: rifampicin HR (95% CI) 0.75, (0.46–1.25), *p* = 0.27; fosfomycin HR (95% CI) 0.72 (0.32–1.62), *p* = 0.43; osteoarticular infections: rifampicin HR (95% CI) 0.71 (0.33–1.49), *p* = 0.97; fosfomycin HR (95% CI) 0.68 (0.24–1.91), *p* = 0.71; endocarditis: rifampicin HR (95% CI) 1.16 (0.49–2.75), *p* = 0.73; fosfomycin HR (95% CI) 0.78 (0.22–2.81), *p* = 0.71	NR	NR	NR
Pericàs, 2018 [29]	RCT	8	Imipenem	7	Vancomycin	Age 84 (67–86) vs. 76 (71–80); males 4 (50) vs. 5 (71)	Infectious endocarditis 4(50) vs. 4 (57), complicated bacteremia 4 (50) vs. 3 (43)	MRSA 15/15 (100)	8 (divided into 4 daily doses)	In-hospital 4/8 (50) vs. 0/7 (0); at 12 weeks after study drug completion 4/8 (50) vs. 1/7 (14)	At the end of study 4/8 (50) vs. 3/7 (42)	7-day 8/8 (100) vs. 6/7 (86)	1/8 (13) vs. 2/7 (29); 1 salt overload vs. 1 acute renal failure and leucopenia, 1 acute renal failure
Baron, 1987 [30]	Prospective observational	17	Anti-staphylococcal penicillin	18	Gentamicin + anti-staphylococcal penicillin	NR	BSI 15/17 (88) vs. 17/18 (94); localized infection 2/17 (12) vs. 1/18 (6)	MSSA 35/35 (100)	[Mean: 237 mg/kg/d; 60 min infusion]	1/17 (6) vs. 1/18 (6)	16/17 (94) vs. 14/18 (78)	NR	1/17 (6) vs. 7/18 (39), *p* < 0.001 (hypokalemia 3, renal damage 1 vs. renal damage 2, superinfection 2, renal impairment (hemodynamic origin) 1, relapse 1)

Abbreviations: *A. baumannii*, *Acinetobacter baumannii*; ADE, adverse drug event; APACHE, Acute Physiology And Chronic Health Evaluation; aHR, adjusted hazard ratio; AKI, acute kidney injury; ALT, alanine transaminase; aOR, adjusted odds ratio; AST, aspartate transaminase; *A. xylosoxidans, Achromobacter xylosoxidans; B. cepacia*, *Burkholderia cepacia*; BLI, β-lactamase inhibitor; BL, β-lactam; *B. catarrhalis*, *Branhamella catarrhalis*; BSI, bloodstream infection; BUN, blood urea nitrogen; *C. freundii*, *Citrobacter freundii*; CI, confidence interval; CK, creatine kinase; *C. koseri*, *Citrobacter koseri*; CLABSI, central line-associated bloodstream infection; CNS, central nervous system; COPD, chronic obstructive pulmonary disease; CRAB, carbapenem-resistant *Acinetobacter baumannii; E. aerogenes*, *Enterobacter aerogenes*; *E. cloacae*, *Enterobacter cloacae*; *E. coli*, *Escherichia coli*; *E. faecalis*, *Enterococcus faecalis*; *E. faecium*, *Enterococcus faecium*; EOT, end-of-treatment; GI, gastrointestinal tract infection; g/d, grams per day; HAP, hospital-acquired pneumonia; *H. influenzae*, *Haemophilus influenzae*; *H. parainfluenza*, *Haemophilus parainfluenzae;* HR, hazard ratio; IAI, intra-abdominal infection; ICU, intensive care unit; IQR, interquartile range; IPTW, inverse probability of treatment weighting; *K. aerogenes*, *Klebsiella aerogenes*; *K. oxytoca*, *Klebsiella oxytoca*; *K. pneumoniae*, *Klebsiella pneumoniae*; KPC, *Klebsiella pneumoniae* carbapenemase; KPC-*K. pneumoniae*, KPC-producing *Klebsiella pneumoniae;* LRTI, lower respiratory tract infection; MDR, multidrug-resistant; MRSA, methicillin-resistant *Staphylococcus aureus*; MSSA, methicillin-susceptible *Staphylococcus aureus*; *n*, number of patients; N, total number of patients; NR, not reported; *P. aeruginosa*, *Pseudomonas aeruginosa*; PDR, pandrug-resistant; RCT, randomized controlled trial; RR, relative risk; *S. agalactiae*, *Streptococcus agalactiae*; SAPS, Simplified Acute Physiology Score; *S. aureus*, *Staphylococcus aureus*; *S. epidermidis*, *Staphylococcus epidermidis*; *S. marcescens*, *Serratia marcescens*; *S. maltophilia*, *Stenotrophomonas maltophilia*; *S. milleri*, *S. pneumoniae*, *Streptococcus pneumoniae*; *Streptococcus milleri*; *S. viridans*, *Streptococcus viridans*; SD, standard deviation; SSTI, skin and soft tissue infection; TOC, test-of-cure; UTI, urinary tract infection; VAP, ventilator-associated pneumonia; XDR, extensively drug-resistant; y, years. Notes: ^a^ others were: *E. coli* (*n* = 2), *C. koseri* (*n* = 2), *S. maltophilia* (*n* = 1), *E. faecium* (*n* = 1); ^b^ fosfomycin combinations group: seven had fluid overload and hypernatremia, and five had hypokalemia; ^c^ lower 30-day mortality in fosfomycin-group statistically significant for KPC-*Klebsiella pneumoniae* (*plog-rank* = 0.02) and not for CRAB (*plog*-*rank* = 0.4); ^d^ 13 received ceftazidime/avibactam monotherapy; ^e^ isolates were detected in 63/75 (84%) patients; ^f^ among the 153 patients with evaluable microbiology outcome; ^g^ daptomycin + fosfomycin group: hypokalemia (*n* = 2), hypocalcemia (*n* = 1), acute renal failure (*n* = 1), creatinine phosphokinase increase (*n* = 1), respiratory tract infection (*n* = 2), acute liver injury (*n* = 1), severe acute digestive bleeding (*n* = 1), nausea/vomiting (*n* = 2), daptomycin alone group: acute renal failure (*n* = 1), creatinine phosphokinase increase (*n* = 1), respiratory failure (*n* = 1), respiratory tract infection (*n* = 1).

**Table 2:** Characteristics and outcomes of patients treated with intravenous fosfomycin monotherapy vs. other antibiotic(s).

Author, Year	Type of Study	N Fosfomycin	N: *n* Comparator	Population Characteristics Total or Fosfomycin Group vs. Comparator Group Median (IQR or Range) or Mean ± SD *n*/N (%)	Infection(s) Total or Fosfomycin Group vs. Comparator Group *n*/N (%)	Pathogen(s) Total or Fosfomycin Group vs. Comparator Group *n*/N (%)	Fosfomycin Dosage g/d	Mortality Fosfomycin Group vs. Comparator Group *n*/N (%)	Clinical Cure Fosfomycin Group vs. Comparator Group *n*/N (%)	Microbiological Cure Fosfomycin Group vs. Comparator Group *n*/N (%)	Adverse Events Fosfomycin Group vs. Comparator Group *n*/N (%)
Falcone, 2024 [32]; Falcone and Tiseo 2025 [31]	Prospective cohort	22:15 fosfomycin monotherapy ^a^	215 ceftazidime/avibactam + aztreonam	Age 71 (IQR 60–79) y; males 273/343 (69); ICU 144/343 (42); SOFA score 3 (IQR 1–7)	BSI 3/22 (14) vs. 139/215 (65), HAP/VAP 4/22 (18) vs. 34/215 (16), UTI 13/22 (59) vs. 28/215 (13), IAI 1/22 (5) vs. 9/215 (4), SSTI 1/22 (5) vs. 5/215 (2)	237/237 (100) MBL-Enterobacterales	12–24 (divided into 3–4 doses)	30-day 4/22 (18) vs. 48/215 (22), *p* = 0.79	NR	NR	NR
Moreno-Mellado, 2025 [33]	Prospective, multicenter, matched-cohort	155	155: 77 ceftriaxone, 25 fosfomycin, 19 piperacillin/tazobactam, 11 ertapenem, 9 amoxicillin/clavulanic acid, 5 meropenem, 5 ciprofloxacin, 4 others	Age 62 (IQR 46–73) vs. 65 (IQR 49–76) y; males 52/155 (34) vs. 52/155 (34); medical ward 107/155 (69) vs. 108/155 (70); ICU 39/155 (25) vs. 35/155 (23); emergency department 8 (5) vs. 10 (7); surgical ward 1/155 (1) vs. 2/155 (1); CCI 1 (IQR 0–3) vs. 1 (IQR 0–3)	Pyelonephritis 94/155 (61) vs. 92/155 (59), cUTI with bacteremia 69/155 (45) vs. 70/155 (45), cystitis 31/155 (20) vs. 27/155 (17), not localizable UTI 8/155 (5) vs. 15/155 (10), renal abscess 3/155 (2) vs. 4/155 (3), cUTI associated with a device 14/155 (9) vs. 13/155 (8), cUTI with hydronephrosis 10/155 (7) vs. 5/155 (3), cUTI with other features 5/155 (3) vs. 4/155 (3)	*E. coli* 310 (100)	16 (124 pts); 12 (23 pts); 8 (8 pts)	30-day 3/155 (2) vs. 9/155 (6), *p* = 0.08	145/155 (94) vs. 140/155 (90), *p* = 0.30	NR	Severe 3/155 (2) vs. 1/155 (1), *p* = 0.34; non-severe 36/155 (23) vs. 12/155 (8), *p* < 0.001
Sojo-Dorado, 2022 [34]	RCT	70 ^b^	73: 31 ceftriaxone, 42 meropenem ^c^	Age 69 (IQR 62–81) vs. 73 (IQR 62–84) y; males 36 (51) vs. 34 (47)	Community-acquired infection 33 (47) vs. 39 (53), health care–associated infection 25 (36) vs. 23 (32), nosocomial infection 12 (17) vs. 11 (15)	*E. coli*	16 (over 60 min)	30-day 2/61 (3) 2/71 (3), *p* = 0.44	59/61 (97) vs. 64/71 (90), *p* = 0.05 ^d^	48/58 (83) vs. 59/69 (86), *p* = 0.33 ^e^	44/70 (63) vs. 41/73 (56), *p* = 0.41; serious 13/70 (19) vs. 10/73 (14), *p* = 0.42
Kaye, 2019 [35]	RCT	184	178 piperacillin/tazobactam	Age 49.9 ± 20.9 vs. 51.3 ± 20.7 y; males 65/184 (35) vs. 67/178 (38); CCI 1 (IQR 0–3) vs. 2 (IQR 1–3), Pitt score 1 (IQR 0–1.25) vs. 1 (IQR 0–2)	Acute pyelonephritis 100/184 (54) vs. 96/178 (54), cUTI 84/184 (46) vs. 82/178 (46)	*E. coli* 133/184 (72) vs. 133/178 (75), *K. pneumoniae* 27/184 (15) vs. 25/178 (14), Enterobacterales 10/184 (5) vs. 9/178 (5), *E. cloacae* species complex 9/184 (5) vs. 3/178 (2), *P. aeruginosa* 8/184 (4) vs. 9/178 (5), *P. mirabilis* 9/184 (5) vs. 5/178 (3), *E. faecalis* 3/184 (2) vs. 7/178 (4), *K. oxytoca* 3/184 (2) vs. 2/178 (1), *C. amalonaticus/farmeri* 1/184 (1) vs. 0/178 (0), *R. ornithinolytica* 1/184 (1) vs. 1/178 (1), *S. marcescens* 1/184 (1) vs. 1/178 (1), *M. morganii* 0/184 (0) vs. 1/178 (1), *A. baumannii-calcoaceticus* species complex 2/184 (1) vs. 0/178 (0), *S. aureus* 1/184 (1) vs. 0/178 (0), *S. saprophyticus* 0/184 (0) vs. 1/178 (1)	18 (over 60 min)	NR	TOC (day 19–21) 121/184 (66) vs. 100/178 (56)	TOC (day 19–21) 167 (91) vs. 163 (92)	99/233 (43) vs. 74/231 (32) ^f^

Abbreviations: *A. baumannii*, *Acinetobacter baumannii*; BSI, bloodstream infection; *C. amalonaticus/farmeri*, *Citrobacter amalonaticus* and *Citrobacter farmeri*; CCI, Charlson Comorbidity Index; cUTI, complicated urinary tract infection; *E. cloacae*, *Enterobacter cloacae*; *E. coli*, *Escherichia coli*; *E. faecalis*, *Enterococcus faecalis*; g/d, grams per day; HAP, hospital-acquired pneumonia; IAI, intra-abdominal infection; ICU, intensive care unit; IQR, interquartile range; IV, *intravenous*; *K. oxytoca*, *Klebsiella oxytoca*; *K. pneumoniae*, *Klebsiella pneumoniae*; MBL, *metallo-β-lactamase; M. morganii*, *Morganella morganii*; n, number of patients; N, total number of patients; NR, not reported; pts, patients; *P. aeruginosa*, *Pseudomonas aeruginosa*; *P. mirabilis*, *Proteus mirabilis*; pts, patients; RCT, randomized controlled trial; *R. ornithinolytica*, *Raoultella ornithinolytica*; *S. aureus, Staphylococcus aureus*; *S. marcescens*, *Serratia marcescens*; *S. saprophyticus*, *Staphylococcus saprophyticus*; SD, standard deviation; SOFA, Sequential Organ Failure Assessment; SSTI, skin and soft tissue infection; TOC, test-of-cure; UTI, urinary tract infection; VAP, ventilator-associated pneumonia, y, years. Notes: ^a^ Seven patients received fosfomycin combination (5 tigecycline, 1 meropenem, 1 gentamicin)]; ^b^ after 4 days IV treatment was discontinued; 60 patients received per os fosfomycin in the fosfomycin group, and 48 patients received oral antibiotics in the comparator group, all based on in vitro AST data; ^c^ when the isolates were ceftriaxone-resistant; ^d^ clinically evaluable population; ^e^ microbiologically evaluable population; ^f^ safety population.

**Table 3:** Characteristics and outcomes of patients treated with combination regimens (including intravenous fosfomycin).

Author, Year	Type of Study	N Combination (*n* Receiving Fosfomycin)	N Comparator (*n* Receiving Fosfomycin)	Infection type(s) Total or Combination vs. Comparator *n*/N (%)	Pathogen(s) Total *n*/N (%)	Mortality Combination vs. Comparator *n*/N (%)	Clinical Cure Combination vs. Comparator *n*/N (%)	Microbiological Cure Combination vs. Comparator *n*/N (%)
Bavaro, 2023 [36]	Retrospective cohort	43 cefiderocol (20)	75 colistin (22)	Primary BSI or UTI 16/43 (37) vs. 21/75 (28), CVC-related 11/43 (26) vs. 25/75 (33), IAI 3/43 (7) vs. 16/75 (21), pneumonia 7/43 (16) vs. 5/75 (7), SSTI 6/43 (14) vs. 6/75 (8), endovascular infection 0/43 (0) vs. 1/75 (1), osteoarticular infection 0/43 (0) vs. 1/75 (1); CCI 6 (IQR 4–8) vs. 6 (IQR 4–7), Pitt score > 4 10/43 (23) vs. 20/75 (27)	CRAB 118/118 (100)	30-day all-cause 17/43 (40) vs. 44/75 (59), *p* = 0.045; 30-day infection related 13/43 (30) vs. 42/75 (56), *p* = 0.007; 90-day all-cause 19/43 (42) vs. 48/75 (64), *p* = 0.032	26/43 (46) vs. 31/75 (54)	NR
Calò, 2023 [37]	Retrospective/prospective observational	11 cefiderocol (4)	29 cefiderocol monotherapy	Primary or CVC-related BSI 5/11 (46) vs. 13/29 (45), pneumonia 5/11 (46) vs. 11/29 (38), SSTI 1/11 (9) vs. 1/29 (3), UTI 0/11 (0) vs. 1/29 (3), bone infection 0/11 (0) vs. 2/29 (7), IAI 0/11 (0) vs. 1/29 (3) ^a^; CCI 3 (IQR 4) vs. 3 (IQR 3.75), Pitt score 2 (IQR 5) vs. 2 (IQR 4.5), SOFA 5.5 (IQR 6.25) vs. 6 (IQR 1)	CRAB 40/40 (100)	5/11 (46) vs. 14/29 (48), *p* = 0.87	7-day 4/11 (36) vs. 17/29 (59); EOT 6/11 (55) vs. 21/29 (72)	7-day 10/11 (91) vs. 22/29 (76); EOT 11/11 (100) vs. 25/29 (86)
Falcone, 2024 [32]; Falcone and Tiseo 2025 [31]	Prospective observational	37 other active antibiotics (OAA) (22) [15 fosfomycin monotherapy] 26 colistin (20) 33 cefiderocol (15)	215 ceftazidime-avibactam/aztreonam (23)	BSI 199/343 (58), HAP/VAP 60/343 (17), UTI 60/343 (17), IAI 13/343 (4), SSTI 11/343 (3); SOFA score 3 (IQR 1–7)	MBL-producing Enterobacterales (344/344) [NDM-producing Enterobacterales: *K. pneumoniae* 326, *E. coli* 2; VIM-producing Enterobacterales: *K. pneumoniae* 5, *E. cloacae* 4, *K. aerogenes* 2, *C. freundii* 2, *E. bugandensis* 1, *Providentia stuartii* 1, *E. coli* 1]	30-day 3/37 (13.5) (OAA), 11/33 (33) (cefiderocol), 13/26 (50) (colistin) vs. 48/215 (22) ^b^	NR	NR
Dalfino, 2023 [38]	Prospective observational	40 cefiderocol + inhaled colistin (21)	50 colistin (0)	VAP 90/90 (100); CCI 5 (IQR 2–6) vs. 7 (IQR 2–8)	CRAB 90/90 (100)	14-day 4/40 (10) vs. 19/50 (38), *p* = 0.03	30/40 (75) vs. 26/50 (52), *p* = 0.02	25/35 (70) vs. 16/40 (40) *p* = 0.003 ^c^
Mazzitelli, 2023 [40]	Retrospective cohort	60 cefiderocol (8)	51 colistin (3)	BSI 34/60 (57) vs. 19/51 (37), pneumonia 26/60 (43) vs. 32/51 (63); APACHE 10 (IQR 7–13) vs. 10 (IQR 7.8–13.2); SOFA 2 (IQR 1–4) vs. 3.5 (IQR 2–5)	CRAB 111/111 (100)	30-day all-cause 26/60 (43) vs. 22/51 (43), *p* = 0.13	44/60 (73) vs. 34/51 (67), *p* = 0.44	26/60 (43) vs. 21/51 (41), *p* = 0.82
Russo, 2023 [41]	Retrospective cohort	19 cefiderocol (14)	54 colistin (5)	VAP + concomitant positive blood cultures 73/73 (100); CCI 2.6 (IQR 1.25–3.75) vs. 2.9 (IQR 1–4); SOFA 9 (IQR 9–10) vs. 10 (9–11)	CRAB 73/73 (100)	14-day 1/19 (5) vs. 41/54 (76), *p* < 0.001; 30-day 6/19 (32) vs. 53/54 (98), *p* < 0.001 ^d^	NR	NR
Falcone, 2022 [39]	Retrospective cohort	47 cefiderocol (8)	77 colistin (5)	BSI 27/47 (57) vs. 52/77 (68), VAP 12/47 (26) vs. 23/77 (30), other 8/47 (17) vs. 2/77 (3); APACHE II 18 (IQR 9–25) vs. 16 (IQR 11–22), CCI 3 (IQR 1–5) vs. 3 (IQR 1–5); SOFA 9 (IQR 6–11) vs. 9 (IQR 4–11)	CRAB 124/124 (100)	30-day 16/47 (34) vs. 43/77 (56), *p* = 0.02	NR	38/46 (83) vs. 69/74 (93) ^e^
Tumbarello, 2021 [42]	Retrospective cohort	412 ceftazidime/avibactam (92)	165 ceftazidime/avibactam monotherapy	BSI 391/577, (68) cUTI 71/577 (12), LRTI 59/577 (10), IAI 35/577 (6), others 21/577 (4); CCI ≥ 3 339/412 (82) vs. 150/165 (91)	KPC-producing *K. pneumoniae* 577/577 (100)	30-day 103/412 (25) vs. 43/165 (26), *p* = 0.79	NR	NR
Zheng, 2021 [43]	Retrospective cohort	41 ceftazidime/avibactam (6)	21 ceftazidime/avibactam monotherapy	BSI 7/41 (17) vs. 2/21 (10), RTI 14/41 (34) vs. 11/21 (52), IAI 9/41 (22) vs. 3/21 (14), UTI 7/41 (17) vs. 4/21 (19), others 4/41 (10) vs. 1/21 (5); APACHE II 18 (IQR 14–20.5) vs. 17 (IQR 16–19), CCI 4 (IQR 3–5) 4 vs. (IQR 3.5–6)	Carbapenem-resistant *K. pneumoniae* 62/62 (100)	30-day 10/41 (24) vs. 11/21 (52) ^f^	NR	30-day 25/41 (61) vs. 9/21 (43)
Khawcharoenporn, 2018 [44]	Retrospective cohort	40 active combined two-drug therapy (22)	74 active monotherapy (12) 22 inactive therapy (2)	HAP/VAP 40/40 (100) vs. 74/74 (100) (active monotherapy), 22/22 (100) (inactive therapy); APACHE II 15 IQR (11–18) vs. 17 (IQR 13–24) (active monotherapy), 16 (IQR 12–26) (inactive therapy)	XDR *P. aeruginosa*	28-day 36/40 (90) vs. 38/74 (51) (active monotherapy), 0/22 (0) (inactive therapy) ^g^	NR	EOT 36/40 (90) vs. 40/74 (54) (active monotherapy), 0/22 (0) (inactive therapy)

Abbreviations: APACHE, Acute Physiology And Chronic Health Evaluation; BSI, bloodstream infection; CCI, Charlson Comorbidity Index; CI, confidence interval; CRAB, carbapenem-resistant *Acinetobacter baumannii*; cUTI, complicated urinary tract infection; CVC-related BSI, central venous catheter-related bloodstream infection; EOT, end of treatment; HR, hazard ratio; IAI, intra-abdominal infection; *K. pneumoniae*, *Klebsiella pneumoniae;* KPC, *Klebsiella pneumoniae* carbapenemase; LRTI, lower respiratory tract infection; N, number of patients; n, number of patients in a subset; NR, not reported; P, *p*-value; RTI, respiratory tract infection; SOFA, Sequential Organ Failure Assessment; SSTI, skin and soft tissue infection; UTI, urinary tract infection; VAP, ventilator-associated pneumonia. Notes: ^a^ data available for 37 patients that had 40 episodes of infection; ^b^ ceftazidime-avibactam/aztreonam-containing regimens and the group of patients receiving other active antibiotics (OAA) were independently associated with 30-day survival in a Cox regression multivariable analysis [adjusted HR (95% CI) 0.33 (0.18–0.62), *p* < 0.001 and 0.35 (0.12–0.98), *p* = 0.05, respectively]; of note: patients in the OAA group receiving fosfomycin-containing regimens (22/33) were less frequently hospitalized in the ICU (4.5% vs. 46%, *p* < 0.001, had less commonly BSI 13.6% vs. 64.7%, *p* < 0.001), and had a lower SOFA score at the time of infection diagnosis (median [interquartile range], 2 [1–3.25] vs. 3 [1,2,3,4,5,6,7]; *p* < 0.001) compared to the ceftazidime-avibactam/aztreonam group; ^c^ microbiological failure was not evaluable in 15 patients (10 in the colistin group and 5 in the cefiderocol group), due to a switch to second-line agents; ^d^ cefiderocol-containing regimens [HR (95% CI) 0.34 (0.18–0.56), *p* < 0.001] and cefiderocol plus fosfomycin combination therapy [HR (95% CI) 0.22 (0.1–0.55), *p* < 0.001] were independently associated with 30-day survival; ^e^ in 120/124 (97%) of patients with available microbiological data; ^f^ combination therapy was significantly associated with lower 30-day mortality [HR (95% CI) 0.167 (0.060–0.465), *p* = 0.001]; ^g^ active.

**Table 4:** Characteristics and outcomes of patients treated with intravenous fosfomycin combination regimens without a comparison group.

Author, Year	Type of Study	N	Population Characteristics Mean ± SD or Median (IQR or Range) *n* (%)	Infection(s) *n* (%)	Pathogen(s) *n* (%)	Resistance *n* (%)	Fosfomycin IV Dosage g/d	Companion to IV Fosfomycin *n* (%)	Mortality *n* (%)	Clinical Cure *n* (%)	Microbiological Cure *n* (%)	Adverse Events *n* (%)
Meschiari, 2024 [45]	Retrospective cohort	70	Age 69 (IQR 61–73) y; males 57 (81.4); ward: ICU 18 (25.7), rehabilitation or post-ICU 23 (32.9), medical 8 (11.4), COVID-19-ICU 6 (8.6), surgical 3 (4.3); CCI 4 (IQR 3–6); SOFA 8 (IQR 6–14); comorbidities: DM 25 (35.7), CHF/CHD 22 (31.4), COVID-19 21 (30.0), renal disease 12 (17.1), solid tumor 12 (17.1), chronic lung disease/COPD 7 (10.0), solid organ transplant 6 (8.6), liver cirrhosis 5 (7.1), hematologic malignancy 2 (2.9)	VAP 16 (22.9), HAP 14 (20.0), VAP/HAP 30 (42.9), osteomyelitis/PJI 12 (17.1), IAI 8 (11.4), cSSTI 8 (11.4), primary BSI 1 (1.4), CLABSI 3 (4.3), cUTI/prostatitis 5 (7.1), meningitis/CNS infection 3 (4.3)	*E. coli* 10 (14.3), *K. pneumoniae* 16 (22.9), *P. aeruginosa* 40 (57.1), *K. aerogenes* 1 (1.4), *P. mirabilis* 1 (1.4), *S. marcescens* 1 (1.4), *C. freundii* 1 (1.4)	ESBL 10 (14.3), AmpC 15 (21.4), carbapenem-resistant 38 (54.3), carbapenem-resistant (non-carbapenemase producers) 22 (31.4), KPC 10 (14.3), VIM 3 (4.3), NDM 3 (4.3), ceftazidime/avibactam resistant 22 (31.4), ceftolozane/tazobactam resistant 20 (27.8)	24 [39 (55.7) pts], 18 [5 (7.1) pts], 16 [26 (37.1) pts]; different dose regimens, based on the severity of the patient’s clinical condition, site of infection, and in vitro sensitivity	Ceftazidime/avibactam 21 (30), meropenem 14 (20), cefiderocol 8 (11.4), piperacillin/tazobactam 6 (8.6), ceftolozane/tazobactam 5 (7.1), tigecycline 4 (5.7), imipenem 2 (2.9), meropenem/vaborbactam 2 (2.9), amikacin 1 (1.4), colistin 1 (1.4)	30-day 11 (15.7), 90-day 22 (31.4)	39 (55.7)	33 (47.1)	Skin reactions 2 (2.9), gastrointestinal reactions 3 (4.3), development of fosfomycin resistance 7 (10)
Russo, 2024 [46]	Retrospective cohort	102	Age 62 (IQR 58.3–71.8); males 78 (76.5); ICU 92 (90.2); CCI 3 (IQR 1–4.75), SOFA 10 (IQR 10–11); comorbidities: CVD 52 (51), DM 48 (47.1), solid tumor 18 (17.6), COPD 10 (9.8), HF 6 (5.9), CKD 4 (3.9), hematological malignancies 4 (3.9)	VAP 60 (58.8), primary BSI 22 (21.6), CVC related 16 (15.6), IAI 2 (2), UTI 1 (1), SSTI 1 (1)	*A. baumannii* 102/102 (100)	CRAB 102/102 (100)	Loading dose of 8 g followed by 12–24 (divided into doses every 6–8 h)	Cefiderocol 54 (52.9), colistin 48 (47.1), ampicillin/sulbactam 18 (17.6)	30-day 48 (47.1), 15-day 32 (31.4)	44 (43.1)	NR	NR
Aysert-Yildiz, 2023 [47]	Retrospective cohort	94	Age 69 (IQR 60–76) y; males 55 (57.9); ICU 52 (54.7); CCI 5 (IQR 4–8); comorbidities: sepsis/septic shock 49 (52.1), solid tumor 41 (43.2), CVD 39 (41.1), DM 39 (41.1), chronic neurological disease 34 (35.8), CKD 24 (25.3), COPD 15 (15.8), chronic hepatic disease 8 (8.4), rheumatic diseases 6 (6.3), hematologic malignancy 2 (2.1)	UTI 27 (28.4), HAP/VAP 25 (26.3), BSI 19 (20.0), IAI 10 (10.5), SSTI 9 (9.5), PJI 2 (2.1), empyema 3 (3.2)	*K. pneumoniae* 94/94 (100)	Resistant to: quinolones 94/94 (100), cotrimoxazole 78/94 (82.9), aminoglycosides 55/94 (58.5), colistin 52/85 (61.2), tigecycline 34/41 (82.9), fosfomycin 10/28 (35.7), ceftazidime/avibactam 7/21 (33.3)	12–24 (divided into 2–3 daily doses); 20–24 in patients with sepsis/septic shock	Most frequently combined with meropenem, polymyxins, or tigecycline; combination with other antimicrobials 87 (92.5); combination with ≥2 antimicrobials 42 (44.7); meropenem-containing regimens 55 (58.5), polymyxin-containing regimens 44 (46.8), tigecycline-containing regimens 20 (21.3) ^a^	30-day 31 (33)	70/93 (75.3) ^b^	55/86 (63.8) ^c^	46 (48.9); leading to discontinuation of therapy 3 (3.2); hypokalemia 35 (37.2), hypernatremia 21 (22.3), elevated LFTs 10 (10.6), hypomagnesemia 8 (8.5), thrombocytopenia 8 (8.5), diarrhea 5 (5.3), eosinophilia 2 (2.1), neutropenia 1 (1.1)
Önal, 2023 [48]	Retrospective cohort	62	Males 23 (37.1); ward: 62 (100); comorbidities: HTN 32 (51.6), DM 28 (45.2), immunosuppression 20 (32.3), malignancy 19 (30.6)	BSI 33 (53.2), VAP 29 (46.8)	*K. pneumoniae* 62 (100)	Carbapenem-resistant 62 (100) *K. pneumoniae*	Patients without 30-day mortality: mean ± SD daily dose 12.08 ± 0.69; patients with 30-day mortality: mean ± SD daily dose 12.37 ± 0.92	Meropenem + colistin/polymyxin B 14 (22.6), meropenem + amikacin/gentamicin 9 (14.5), meropenem 12 (19.4), colistin/polymyxin B 7 (11.3), amikacin/gentamicin 3 (4.8), colistin/polymyxin B + amikacin/gentamicin 2 (3.2), two others ^d^ 10 (16.1), others ^d^ 5 (8.1),	14-day all-cause 22 (36.5); 30-day all-cause 34 (54.8)	NR	NR	At 30 days: hypernatremia 34 (54.8), hypokalemia 34 (54.8)
Gatti, 2022 [50]	Retrospective case series	6	Age 57.7 ± 21.7 y; males 3 (50); ward: ICU 4 (66.7), infectious disease unit 1 (16.7), hematology + ICU 1 (16.7)	BSI + VAP 2 (33.3), VAP 2 (33.3), BSI 1 (16.7), HAP 1 (16.7)	*P. aeruginosa* 6 (100)	DTR *P. aeruginosa* 6 (100): ceftazidime/avibactam-resistant 4 (66.7), ceftolozane/tazobactam-resistant 2 (33.3)	16–24 (continuous infusion; 8 g loading dose)	Cefiderocol 3 (50), ceftazidime/avibactam 2 (33.3), cefiderocol 1 (16.7)	Total 30-day 2 (33.3)	NR	5 (83.3)	0 (0)
Thampithak, 2022 [51]	Retrospective descriptive	254	Age 59.6 ± 17.7 y; males 142 (53.6); ward: critical care 26 (9.8), general internal medicine 163 (61.5), critical surgery 8 (3.0), surgical 68 (25.7); comorbidities: CKD 147 (55.5), HTN 62 (23.4), DM 53 (20.0), CVD 39 (14.7), solid tumor 24 (9.1), hematologic malignancy 22 (8.3) (these values refer to a total of 265 patients who received IV fosfomycin; 11 received it as surgical prophylaxis and 254 received it for the treatment of infections)	RTI 118 (46.5), UTI 53 (20.9), SSTI 29 (11.4), BSI 24 (9.4), IAI 7 (2.8), CNS infection 1 (0.4), BJI 1 (0.4), unknown source 21 (8.3)	Enterobacterales 125 (47.2), *A. baumannii* 116 (43.8), *P. aeruginosa* 24 (9.1)	Carbapenem-resistant 141 (87.6)	Based on the infection site: RTI 2–16, UTI 2–12, SSTI 1–12, BSI 2- 12, IAI 2–12, febrile neutropenia 8–12, CNS infection 8, BJI 4	Colistin 132, aminoglycosides 41, tigecycline 2, carbapenem 8, levofloxacin 4, ampicillin/sulbactam 2, ceftazidime 2, colistin–ampicillin/sulbactam 17, colistin–carbapenem 12, colistin–aminoglycosides 3, colistin–levofloxacin 7, colistin–piperacillin/tazobactam 1, colistin–tigecycline 1, colistin–cotrimoxazole 1, aminoglycosides–tigecycline 1, aminoglycosides–meropenem 2, meropenem–levofloxacin 2, meropenem–piperacillin/tazobactam 1, aminoglycosides–cotrimoxazole 1 ^e^	14-day all-cause 119 (45) ^f^	NR	NR	NR
Abdallah, 2021 [52]	Retrospective cohort	30	Age 63.5 (IQR 46–73) y; males 19 (63.3); CCI 6 (IQR 3.8–9); comorbidities: recent hospitalization 23 (76.6), DM 19 (63.3), CKD 16 (53.3), history of recurrent UTIs 14 (46.6), malignancy 11 (36.7), recent surgery 10 (33.3), transplant recipient 5 (16.7), CHD 2 (6.6), chronic lung disease 2 (6.6)	UTI 17 (56.7), BSI 4 (13.3), SSTI 4 (13.3), IAI 2 (6.7), RTI 2 (6.7), CSF infections 1 (3.3)	*K. pneumoniae* 17 (56.7), *E. coli* 7 (23.3), other 6 (20)	DTR Gram-negative bacteria 30 (100)	12–24 g (divided into 2–4 daily doses)	Meropenem 8 (26.6), tigecycline 8 (26.6), aminoglycoside 5 (15.7), colistin 3 (10), fluoroquinolone 3 (10) ^g^	Total 30-day 7 (23.3)	22 (73.3)	20 (66.7)	Most frequent adverse events: hypokalemia 13 (43.3), hypernatremia 7 (23.3), resistance to fosfomycin within 90 days of initiation of fosfomycin therapy 5 (16.7)
Ballouz, 2021 [53]	Retrospective cohort	28	Age 56.43 ± 17.66 y; males 20 (71); comorbidities: hematologic 9 (32), HTN 9 (32), dyslipidemia 7 (25), DM 5 (18), acute myeloid leukemia 4 (14), coronary artery disease 3 (11), colorectal cancer 2 (7), diffuse large B-cell lymphoma 2 (7), acute lymphoblastic leukemia 1 (4), bladder cancer 1 (4), chronic lung disease 1 (4), CKD 1 (4), COPD 1 (4), Hodgkin lymphoma 1 (4), squamous cell carcinoma of the mandible 1 (4), T-cell lymphomas 1 (4)	BSI 18 (64), UTI 5 (16), RTI 3 (10), febrile neutropenia 3 (10)	*E. coli* 11 (40), *A. baumannii* 9 (32), *P. aeruginosa* 4 (14), *Enterobacter* spp. 2 (7), *K. pneumoniae* 2 (7)	*A. baumannii* (MDR 22, XDR 78), *E. coli* (MDR 50, XDR 50), *P. aeruginosa* (MDR 50, XDR 50), *K. pneumoniae* (MDR 64, XDR 27), *Enterobacter* spp. (MDR 25, XDR 50),	12–16 (divided in 2–4 daily doses); higher daily doses (up to 24 g) were used in severe infections	Fosfomycin was administered in combination with other antibiotics 26 (mainly tigecycline 8, amikacin 3, based on susceptibility data) ^h^	Overall in-hospital 7 (26)	EOT 14 (45); out of 31 episodes that were treated	At EOT 8/11 (73; out of the 11 cultures available at the EOT	Hypokalemia (14), hypernatremia (6), diarrhea 5), out of which 4 were due to *C. difficile*; development of resistance to fosfomycin (2)
Perdigão Neto, 2019 [54]	Prospective case series	13	Age 52 ± 24 y; males 8 (61.5); ICU 9 (69.2); comorbidities: recent surgery 8 (61.5), DM 7 (53.8), HTN 7 (53.8), immunosuppression 7 (53.8), dyslipidemia 2 (15.4), Chagas’ disease 1 (7.7), OA 1 (7.7)	CLABSI 6 (46.2), BSI-IAI 3 (23.1), BSI-UTI 1 (7.7), BSI-VAP 1 (7.7), SSI 1 (7.7), VAP 1 (7.7)	*K. pneumoniae* 9 (69.2), *S. marcescens* 3 (23.1), *P. aeruginosa* 1 (7.7)	KPC-2 10 (76.9), CTX-M 9 (69.2), SHV 9 (69.2), TEM 8 (61.5), OXA 5 (38.5), NDM-1 1 (7.7), PAO 1 (7.7)	16 (divided in 4 daily doses)	Meropenem 10 (76.9), amikacin 3 (23.1), tigecycline 2 (15.4), colistin 2 (15.4), ertapenem 1 (7.7); some received more than one agent	14-day 5 (38.5), 28-day 7 (53.8)	8 (61.5)	NR	8 (61.5); some patients had more than one (hypokalemia 8, nausea 3, vomiting 2, diarrhea 2, hypertension 2)
Bodmann, 2025 [55]	Prospective non-interventional	716	Age 62.8 ± 14.75 y; males 454 (63.4); ICU 370 (51.7); comorbidities: cardiovascular 476 (66.5), renal 253 (35.3), endocrinologic 252 (35.2), electrolyte disorders 226 (31.6), respiratory 210 (29.3), immunocompromised 163 (22.8), oncologic 128 (17.9), immunosuppressive 120 (16.8), hepatic 109 (15.2), orthopedic 100 (14.0), traumatic injury/fractures 60 (8.4), other 273 (38.1)	BSI 169 (23.6), cUTI 129 (18), BJI 124 (17.3), HAP/VAP 79 (11), cSSTI 65 (9.1), BM/CNS 56 (7.8), IE 46 (6.4)	*S. aureus* 225 (31.4), *Klebsiella* spp. 123 (17.2), *E. coli* 102 (14.2), CoNS 93 (13), other Enterobacterales 78 (10.9), *P. aeruginosa* 60 (8.4), *Enterococcus* spp. 58 (8.1), *Streptococcus* spp. 32 (4.5), other Gram-negative pathogens 15 (2.1), other Gram-positive pathogens 12 (1.7), anaerobes 8 (1.1), *Acinetobacter* spp. 7 (1)	MDR 248 (34.6) [methicillin resistance 53 (7.4), vancomycin-resistant 13 (1.8), carbapenem-resistant 79 (11), ESBL-producing 62 (8.7), other mechanism of resistance 75 (10.5)]; some isolates had more than one mechanism of resistance	Median daily targeted dose of 15 (with targeted doses up to 24 g); median daily targeted dose: 15 in Germany and the UK, 16 in Italy, 20 in Greece, 24 in Austria	BL/BLIs 181 (25.3), carbapenems 157 (21.9), penicillins 109 (15.2), 3rd/4th/5th/next-generation cephalosporins 80 (11.2), 1st/2nd-genenration cephalosporins: 79 (11), daptomycin 56 (7.8), fluoroquinolones 37 (5.2), aminoglycoside 28 (3.9), linezolid 23 (3.2), colistin 16 (2.2)	Overall in-hospital 76 (10.6); none was related to treatment with fosfomycin	539 (75.3) [clinical response 597 (83.4)]	590 (82.4)	417 (58); serious 59 (8) [hypokalemia 189 (26.4), hypernatremia 109 (15.2)]
Luciano, 2025 [56]	Retrospective cohort	56	Age 62.4 (IQR 30–75) y; males 38 (68); ward: internal medicine 27 (48), ICU 17 (30), cardio-thoracic surgery unit 6 (11), respiratory disease unit 4 (7), cardiovascular disease unit 2 (4); CCI 4 (IQR 3–6); comorbidities: renal disease 17 (30.4), DM 16 (28.6), heart disease 15 (26.8), HTN 13 (23.2)	RTI 23 (41.1), BSI 11 (19.6), bone infections 9 (16.1), UTI 9 (16.1), surgical site infections 4 (7.1)	*P. aeruginosa* 14 (17.3), *E. coli* 12 (14.8), *K. pneumoniae* 11 (13.6), *S. aureus* 7 (8.6), *A. baumannii* 5 (6.2), *E. faecium* 4 (4.9), *S. epidermidis* 4 (4.9), *S. hominis* 4 (4.9), *E. cloacae* 3 (3.7), *E. faecalis* 2 (2.5), *K. aerogenes* 2 (2.5), S. *haemolyticus* 2 (2.5), *S. maltophilia* 2 (2.5), C. *striatum* 1 (1.2), *E. raffinosus* 1 (1.2), *H. influenzae* 1 (1.2), *H. alvei* 1 (1.2), *K. oxtytoca* 1 (1.2), *M. catarrhalis* 1 (1.2), *P. putida* 1 (1.2), *S. simulans* 1 (1.2), *S. paucimobilis* 1 (1.2)	MDR 36 (64.3) [methicillin-resistant 14 (25), ESBL 8 (14.3), DTR 4 (7.1), KPC 4 (7.1), XDR 4 (7.1), AmpC 1 (1.8)], AmpR 1 (1.8)]	NR	Meropenem 16 (28.6), colistin 11 (19.6), ceftazidime/avibactam 9 (16.1), daptomycin 5 (8.9) cefiderocol 5 (8.9), ceftolozane/tazobactam 4 (7.1), ceftobiprole 4 (7.1), linezolid 4 (7.1), tigecycline 4 (7.1), piperacillin/tazobactam 3 (5.4), amikacin 2 (3.6), gentamicin 2 (3.6), amoxicillin/clavulanate 2 (3.6), cotrimoxazole 2 (3.6), teicoplanin 2 (3.6), vancomycin 2 (3.6), meropenem/vaborbactam 2 (3.6), other (1 of each: ertapenem, cefepime, metronidazole, ceftaroline, ampicillin/sulbactam, cefazolin) 6 (10.7)	All-cause at EOT 14 (25)	22 (39.3)	25 (69.4); follow-up cultures were performed in 36 patients	Electrolyte imbalance 16 (29.6); neutrophil count reduction: median 8340 to 5730, *p* = 0.01; increase in serum Na^+^ concentration: median 138 to 140 mEq/L, *p* < 0.01
Zerbato, 2025 [57]	Retrospective cohort	393	Age 69 (IQR 59–76) y; males 268 (68.2); ward: ICU 178 (45.3), medical 91 (23.2), surgical 83 (21.1), infectious disease 41 (10.4)	Pneumonia 113 (34.3), BSI 71 (21.7), UTI 70 (21.1), other ^i^	*E. coli* 56 (23), *P. aeruginosa* 55 (22.6), *K. pneumoniae* 43 (17.7), *S. aureus* 32 (13.2), *E. faecium* 12 (4.9), *S. epidermidis* 12 (4.9), A. *baumannii* 3 (1.2), S. *enterica* 1 (0.4) (among the 296 total)	ESBL-producers 32 (10.8), AmpC β-lactamase producers 14 (4.7), KPC-producers 11 (3.7), methicillin-resistant *S. epidermidis* 9 (3), MRSA 8 (2.7), OXA-48-producers 7 (2.4), VRE 7 (2.4), XDR isolates 6 (2), MBL producers 2 (0.7),	2–24 (continuous infusion in 192 patients)	Piperacillin/tazobactam 82 (20.9), new β-lactamase inhibitor combination (ceftazidime/avibactam, ceftolozane/tazobactam, or meropenem/vaborbactam) 71 (18.1), carbapenem 70 (17.8), daptomycin 42 (10.6), tigecycline 31 (8.0), linezolid 27 (6.8), vancomycin 22 (5.6), anti-pseudomonal cephalosporin 17 (4.3), aminoglycoside 17 (4.3), third generation cephalosporin 15 (3.8), ceftazidime 10 (2.5), colistin 8 (2.0), others 66 (16.8)	30-day 85 (21.6), 60-day 105 (26.7), 90-day 115 (29.3)	NR	NR	*C. difficile* infection 8 (2)
Anastasia, 2023 [58]	Retrospective cohort	343	Age 68 ± 13.9 (19–95) y; males 216 (62.9); high-ICU 57/343 (16.6); comorbidities: CVD 57 (16.6), lung diseases 57 (16.6), DM 48 (13.9), solid neoplasm 31 (9.1), hematological diseases 29 (8.4), kidney failure 29 (8.4), SARS-CoV-2 18 (5.2), HIV/AIDS 11 (3.2), other 59 (17.2)	UTI/pyelonephritis 69 (20.1), IE 13 (3.8), SSTI 49 (14.3), CNSI 10 (2.9), osteomyelitis 37 (10.8), BSI 52 (15.2), intrathoracic infections 6 (1.7), IAI 37 (10.8), pneumonia 63 (18.4), other 7 (2)	*K. pneumoniae* 193 (56.2), *P. aeruginosa* 42 (12.2), *A. baumannii* 36 (10.2), *Enterococcus* spp. 28 (8.2), *S. aureus* 16 (4.2), other 28 (8)	Resistant K. pneumoniae: cotrimoxazole 164/193 (85.0), carbapenems 159/193 (82.4), amikacin 182/193 (94.3), ceftazolane/tazobactam 78/84 (92.9), colistin 47/193 (24.4), fosfomycin 15/193 (7.8), ceftazidime/avibactam 10/100 (10), meropenem/vaborbactam 0/22 (0); resistant *A. baumannii*: fluoroquinolones 36/36 (100), cotrimoxazole 31/36 (86.1), aminoglycosides 29/36 (80.6), colistin 2/36 (5.6)	16–24 (divided into 3–4 daily doses)	Ceftazidime/avibactam 122 (35.5), meropenem 57 (16.6), colistin 49 (14.1), daptomycin 39 (11.4), vancomycin 28 (8.2) ^j^	90/343 (26.2)	226/343 (65.8)	NR	20 (5.8); nausea 7 (2), isolated hypernatremia 4 (1.2), isolated hypokalemia 4 (1.2), diarrhea 3 (0.9), hypernatremia + hypokalemia 1 (0.3), rash 1 (0.3)
Zhanel, 2023 [49]	Registry-based cohort	51	Ward: ICU 35 (68.6), non-ICU 16 (31.4)	BSI/sepsis 14 (27.5), VAP 10 (19.6), HAP 8 (15.7), cIAI 6 (11.8), cUTI 6 (11.8), BJI 2 (3.3), CABP (3.3), endocarditis 2 (3.3), CNSI 1 (2)	*Klebsiella* spp. 16, *E. coli* 12, *P. aeruginosa* 12, *Enterobacter* spp. 4, *E. faecium* 4, *Citrobacter* spp. 2, *S. aureus* 1, unknown 1; 2 patients had mixed infections (*E. coli* and *Klebsiella* spp. and ESBL *Klebsiella* spp. + *P. aeruginosa*)	CRE 21 (CRE *Klebsiella* 11, CRE *E. coli* 10); VRE 3; ESBL 3 (ESBL *Klebsiella* 2, ESBL-containing mixed infection 1)	2–24 (divided into 1–4 daily doses); 3 patients received 2 g post-dialysis, and 1 patient received an unknown dose	Carbapenem 13, carbapenem + tigecycline 9, aminoglycoside 8, colistin or polymyxin b 4, carbapenem + colistin 3, aminoglycoside + carbapenem 2, carbapenem + fluoroquinolone 1, carbapenem + inhaled colistin 1, carbapenem + piperacillin/tazobactam 1, cefazolin + daptomycin + rifampin 1, ceftazidime + intrathecal colistin 1, daptomycin 1, doxycycline 1, fluoroquinolone + piperacillin/tazobactam 1, imipenem/relebactam 1, tigecycline 1, vancomycin 1	14 (27.5)	13 (25.5) [2 patients had an unknown outcome]	19 (31.1); presumed eradication 15 (29.4) [5 patients had an unknown outcome]	Hypokalaemia 7 (13.7), gstrointestinal 2 (3.92), hypernatraemia 2 (3.92)
Zirpe, 2021 [59]	Retrospective cohort	309	Age 60.59 ± 15.90 y; males 193 (62.5)	BSI 140 (45.3), UTI 53 (13.9), pneumonia 49 (15.9), SS 44 (14.2), SSTI 10 (3.2), IE 4 (1.3), meningitis 2 (0.7), osteomyelitis 2 (0.7), IAI 3 (1), gynecological infection 1 (0.3)	*K. pneumoniae* 149 (48.2), *E. coli* 49 (15.9), *P. aeruginosa* 27 (8.7), *Staphylococcus* spp.12 (3.9), *E. aerogenes* 3 (0.9), *P. mirabilis* 3 (0.9), *S. marcescens* 2 (0.6), mixed 47 (15.2), no growth 17 (5.5)	Suspected CRE infection 1 (0.3)	3- >24	NR	Overall 139 (45)	50 (16.2)	NR	Hypokalemia 190 (61.5), hypernatremia 75 (24.3)
Putensen, 2019 [60]	Prospective non-interventional	209	Age 59.1 ± 16.4 y; males 132 (63.2); ICU 194 (92.8); comorbidities: diabetes mellitus, smoking, immunosuppression/corticosteroids, chronic renal insufficiency, liver cirrhosis, intensive antibiotic pretreatment within the last month	CNS 45 (21.5), pneumonia (CAP, HAP, VAP) 32 (15.3), BJI 23 (11), IAI 23 (11), sepsis/BSI 22 (10.5), endocarditis (all with sepsis/BSI) 9 (4.3), cUTI 8 (3.8); APACHE II (in 71 patients) 23 ± 8, APACHE III (in 19 patients) 104 ± 18	*S. aureus* 58 (22.3), *S. epidermidis*/CoNS 37 (14.2), *E. coli* 32 (12.3), *Enterococcus* spp. 28 (10.8), *Klebsiella* spp. 20 (7.7), *Enterobacter/Citrobacter* spp. 17 (6.5), *P. aeruginosa* 12 (4.6), aerobes 9 (3.5), *Streptococcus* spp. 9 3.5), *Proteus* spp. 6 (2.3), *Serratia* spp. 4 (1.5), other Gram-negative pathogens 19 (7.3), other Gram-positive pathogens 9 (3.6)	At least one MDR pathogen: 51 (24.4); ESBL 4 (1.9); *S. aureus* infections: MSSA 21 (75), MRSA 7 (25)	Total dose range 3–24 [15 g or less per day (166 pts), more than 15 g (41 pts)]	Carbapenem 102 (48.8), glycopeptide 66 (31.6), 3rd- or 4th-generation cephalosporin 58 (27.8), penicillins/BLI 30 (14.4), metronidazole 26 (12.4), quinolone 23 (11.0), penicillin 22 (10.5), 1st or 2nd generation cephalosporin 13 (6.2), linezolid 13 (6.2), aminoglycoside 13 (6.2), macrolide 12 (5.7), sulfonamide 11 (5.3), daptomycin 10 (4.8), colistin 7 (3.3), rifampicin 7 (3.3), tigecycline 6 (2.9), other 9 (4.3)	All-cause 15 (7.2)	148 (81.3) [clinical per protocol (cPP) population, *n* = 182]	63 (70.0) [microbiological per protocol (mPP) population, *n* = 90]	70 (30.1), non-serious 36 (14.8), serious 3 (1.4); hypernatremia 31 (14.8), hypokalemia 13 (6.2), diarrhea 3 (1.4), nausea 2 (1.0), transaminases increased 1 (0.5), pyrexia 1 (0.5), allergic reaction 1 (0.5), hyponatremia 1 (0.5), hyperkalemia 1 (0.5), not specified 1 (0.5)
Chuang, 2022 [61]	Prospective cohort	106	Age 67.8 (IQR 54.6–79.9) y; males 66 (62.3); CCI 3 (IQR 2–5); comorbidities: immunosuppressive use 57 (53.8), steroid use 30 (28.3), DM 26 (24.5), CKD 25 (23.6), CHD 10 (9.43), leukemia 17 (16.0), liver cirrhosis 7 (6.6)	CLABSI 70 (66), UTI 34 (32.1), primary BSI 20 (18.9), IAI 11 (10.4)	*E. faecium* 106 (100)	Vancomycin-resistant *E. faecium*	16 (8–22.5)	Daptomycin 106 (100)	Overall 62 (58.5), 28-day 43 (40.6)	59 (55.7)	80 (75.5)	Elevated CK 11 (10.4), new onset of thrombocytopenia 18 (17), hypernatremia: 15 (14.2), hypokalemia: 49 (46.2)
Coronado-Álvarez, 2019 [62]	Retrospective cohort	75	NR	BSI 75 (100), CRBSI 16 (21.3), IE 29 (38.7), PJI 3 (4)	MRSA 45 (60), MSSA 22 (29.3), *E. faecium* 8 (10.7)	Methicillin-resistant 45 (60)	NR	daptomycin 30, oxacillin 22, vancomycin 15, linezolid 8	NR	61 (81.3)	61 (81.3)	Minor 9 (phlebitis or minor hypernatremia; severe: 1 ^k^
Karnmueng, 2024 [63]	Retrospective cohort	134 (23 received fosfomycin)	Age 63.6 (mean, SD 19.2) y, males 80 (60), ICU 52 (39); APACHE II 20.03 ± 6.5, SOFA 7.04 ± 4.6, Pitt score 2.60 ± 2.2; comorbidities: solid malignancies 47 (35), diabetes mellitus 43 (32), chronic kidney disease 23 (17); CCI 5.38 (mean, SD 3.1), APACHE II 20.72 (mean, SD 6.5), SOFA score 7.04 (mean, SD 4.6), Pitt bacteremia score 2.6 (mean, 2.2 SD), INCREMENT-CPE score 9.86 (mean, 3.8 SD)	BSI catheter-related 32 (24), BSI primary 28 (21), pneumonia 28 (21)	*K. pneumoniae* 115 (86), *E. coli* 16 (12), *E. cloacae* 3 (2)	CPE 60 (45) [NDM-1 + OXA-48 31 (52), OXA-48 19 (32), NDM-1 9 (15)]	NR	NR	Overall all-cause 14-day mortality 47 (35) ^l^	NR	NR	NR

Abbreviations: *A. baumannii*, *Acinetobacter baumannii*; AIDS, acquired immunodeficiency syndrome; AmpC, Ambler Class C β-lactamase; AmpR, Ampicillin-resistant; b, refers to a footnote on clinical cure data; APACHE, Acute Physiology And Chronic Health Evaluation; BJI, bone and joint infection; BL/BLIs, β-lactam/β-lactamase inhibitors; BM, bone marrow; BSI, bloodstream infection; c, refers to a footnote on microbiological cure data; CABP, community-acquired bacterial pneumonia; CAP, community-acquired pneumonia; CCI, Charlson Comorbidity Index; CHF, congestive heart failure; cIAI, complicated intra-abdominal infection; CK, creatine kinase; CKD, chronic kidney disease; CLABSI, central line-associated bloodstream infection; CNS, central nervous system; CNS infection, central nervous system infection; CNSI, central nervous system infection; CoNS, coagulase-negative *Staphylococci*; COPD, chronic obstructive pulmonary disease; cPP, clinical per protocol; CRAB, carbapenem-resistant *Acinetobacter baumannii*; CRE, carbapenem-resistant Enterobacterales; CRBSI, catheter-related bloodstream infection; cSSTI, complicated skin and soft tissue infection; CSF, cerebrospinal fluid; cUTI, complicated urinary tract infection; CTX-M, Cefotaximase-M (a type of ESBL); CVD, cardiovascular disease; CVC-related BSI, central venous catheter-related bloodstream infection; *C. difficile*, *Clostridioides difficile*; *C. freundii*, *Citrobacter freundii*; *C. striatum*, *Corynebacterium striatum*; DM, diabetes mellitus; DTR, difficult to treat; *E. aerogenes*, *Enterobacter aerogenes*; *E. cloacae*, *Enterobacter cloacae*; *E. coli*, *Escherichia coli*; EOT, end of treatment; ESBL, extended-spectrum β-lactamase; *E. faecalis*, *Enterococcus faecalis*; E. faecium, *Enterococcus faecium*; *E. raffinosus*, *Enterococcus raffinosus*; HAP, hospital-acquired pneumonia; HF, heart failure; HIV, human immunodeficiency virus; HTN, hypertension; *H. alvei*, *Hafnia alvei*; *H. influenzae*, *Haemophilus influenzae*; IAI, intra-abdominal infection; ICU, intensive care unit; IE, infective endocarditis; IQR, interquartile range; IV, intravenous; KPC, *Klebsiella pneumoniae* carbapenemase; *K. aerogenes*, *Klebsiella aerogenes*; *K. oxtytoca*, *Klebsiella oxtytoca*; *K. pneumoniae*, *Klebsiella pneumoniae*; LFTs, liver function tests; MBL, metallo-β-lactamase; MDR, multidrug-resistant; *M. catarrhalis*, *Moraxella catarrhalis*; mPP, microbiological per protocol; MRSA, methicillin-resistant *Staphylococcus aureus*; MSSA, methicillin-sensitive *Staphylococcus aureus*; NDM, New Delhi metallo-β-lactamase; NR, not reported; OA, osteoarthritis; OXA, oxacillinase; OXA-48, Oxacillinase-48; PAO, *P. aeruginosa* (often refers to a specific strain); PJI, prosthetic joint infection; *P. aeruginosa*, *Pseudomonas aeruginosa*; *P. mirabilis*, *Proteus mirabilis*; *P. putida*, *Pseudomonas putida*; RTI, respiratory tract infection; S. aureus, *Staphylococcus aureus*; SARS-CoV-2, severe acute respiratory syndrome coronavirus 2; *S. enterica*, *Salmonella enterica*; SHV, Sulfhydryl Variable β-lactamase; SOFA, Sequential Organ Failure Assessment; *S. epidermidis*, *Staphylococcus epidermidis*; S. haemolyticus, *Staphylococcus haemolyticus*; *S. hominis*, *Staphylococcus hominis*; *S. maltophilia*, *Stenotrophomonas maltophilia*; *S. marcescens*, *Serratia marcescens*; *S. paucimobilis*, *Sphingomonas paucimobilis*; S. simulans, *Staphylococcus simulans*; SS, Sepsis/Septic Shock; SSI, surgical site infection; SSTI, skin and soft tissue infection; TEM, Temoniera (a type of β-lactamase); UTI, urinary tract infection; VAP, ventilator-associated pneumonia; VIM, Verona integron-encoded metallo-β-lactamase; VRE, vancomycin-resistant *Enterococci*; XDR, extensively drug-resistant; y, years. Notes: ^a^ seven patients received monotherapy; ^b^ clinical response was not evaluated in one patient due to referral to another hospital; ^c^ among the 86 patients who had subsequent microbiological cultures; ^d^ ceftazidime/avibactam, cefoperazone/sulbactam, tigecycline, co-trimoxazole, ciprofloxacin, and levofloxacin; ^e^ 16 patients received monotherapy; ^f^ the 14-day mortality was higher in patients who received fosfomycin + colistin compared to fosfomycin + aminoglycoside (53.7% vs. 30.6%, *p* = 0.016); ^g^ some patients received more than two antibiotics in combination with fosfomycin and 3 patients received fosfomycin monotherapy; ^h^ 2 patients received monotherapy; ^i^ bone, CNS, genitals, heart, abdomen, prosthetic implants, skin and soft tissue, vascular system infections; ^j^ Empirically, in cases of infections without microbiological findings: meropenem 20, daptomycin 14, vancomycin 8; fosfomycin monotherapy 19/343, fosfomycin combination with other antimicrobial agents 324/343; ^k^ acute cardiogenic pulmonary oedema secondary to sodium overload after 10 days of daptomycin + fosfomycin therapy; ^l^ multivariate Cox proportional-hazards model revealed a protective effect on 14-day mortality of fosfomycin-based regimen.

**Table 5:** Characteristics of patients treated with fosfomycin monotherapy without a comparison group.

Author, Year	Type of Study	N	Population Characteristics Mean ± SD or Median (IQR or Range) or *n* (%)	Infection(s) *n* (%)	Pathogen(s) *n* (%)	Resistance *n* (%)	Fosfomycin IV Dosage g/d	Mortality *n* (%)	Clinical Cure *n* (%)	Microbiological Cure *n* (%)	Adverse Events *n* (%)
Rodríguez-Gómez, 2025 [64]	Retrospective cohort	47	Age 80 (74–86) y; males 21 (9.9); CCI 6 (IQR 4–9); comorbidities: DM 29 (61.7), CRF 20 (42.6), CHF 18 (38.3), hemodialysis 2 (4.3), solid organ transplantation 2 (4.3)	UTI 47 (100)	*K. pneumoniae* 47 (100)	KPC carbapenemase-producing *K. pneumoniae* 47 (100)	16 (divided into 4 daily doses)	30-day all-cause 12 (25.5)	21-day 33 (70.2)	14-day 22 (73.3) (with commercial microdilution; out of 30 patients with post-treatment urine culture obtained)	Hypokalemia 7 (14.9), hyponatremia 6 (12.8)
Falcone, 2024 [32]; Falcone, 2025 and Tiseo [31]	Prospective cohort	15	NR	UTI 12 (80), BSI 1 (6.7), SSTI 1 (6.7), HAP 1 (6.7)	Enterobacterales	MBL-producing 15 (100)	NR	3 (20)	NR	NR	NR
Aysert-Yildiz, 2023 [47]	Retrospective cohort	7	NR	UTI 6 (86), SSTI 1 (14)	*K. pneumoniae* 7 (100)	Carbapenem resistant 7 (100)	12–24 (divided into 2–3 daily doses; infused over at least 30–60 min)	0 (0)	7 (100)	NR	NR
Zhanel, 2023 [49]	Registry-based cohort	8	Ward: ICU 4 (50), non-ICU 4 (50)	cUTI 6 (75), VABP 1 (13), BSI/sepsis 1 (13); 1 patient with cUTI had concomitant BSI/sepsis	*E. coli* 2 (25), *Klebsiella* spp. 2 (25), *P. aeruginosa* 2 (25), *Citrobacter* spp. 1 (12.5), *Enterobacter* spp. 1 (12.5)	NR	4–24 (divided into 2–3 daily doses; all were administered over a period of 15 min–1 h) ^a^	1 (12.5)	5 (62.5); improvement 1, unknown 1)	5 (62.5); (persistence 2, unknown 1)	1 (12.5) elevated liver enzymes
Kanchanasurakit, 2020 [65]	Prospective pilot	8	Age 66.92 ± 7.26 y; males 3 (37.5); comorbidities: type 2 DM 5 (62.5), HTN 4 (50), AF 2 (25), BPH 1 (12.5), COPD 1 (12.5), Ischemic stroke 1 (12.5), Schizophrenia 1 (12.5), stage 3a CKD 1 (12.5), stage 3b CKD 1 (12.5)	UTI with sepsis/septic shock 8 (100)	*K. pneumoniae* 6 (75), *E. coli* 2 (25)	Carbapenem resistant 8 (100)	6–16 (divided into 3–4 daily doses; loading dose 2–4 g; 4–6 h infusion) ^b^	0 (0)	NR	7 (87.5)	Hypokalemia 3 (37.5), hypernatremia (12.5)

Abbreviations: *A. baumannii, Acinetobacter baumannii*; AF, Atrial fibrillation; BPH, Benign prostatic hyperplasia; BSI, Bloodstream infection; CCI, Charlson Comorbidity Index; cUTI, Complicated urinary tract infection; CHF, Congestive heart failure; *Citrobacter* spp., *Citrobacter species*; CKD, Chronic kidney disease; COPD, Chronic obstructive pulmonary disease; CRF, Chronic renal failure; DM, Diabetes mellitus; *E. coli, Escherichia coli*; *Enterobacter* spp., *Enterobacter* species; g/d, Grams per day; HAP, Hospital-acquired pneumonia; HTN, hypertension; ICU, intensive care unit; IQR, interquartile range; IV, intravenous; *K. pneumoniae, Klebsiella pneumoniae*; KPC, *Klebsiella pneumoniae* carbapenemase; *Klebsiella* spp., *Klebsiella* species; LD, loading dose; MBL, Metallo-β-lactamase; NR, not reported; non-ICU, non–intensive care unit; *P. aeruginosa, Pseudomonas aeruginosa*; RTI, respiratory tract infection; SD, standard deviation; SSTI, skin and soft tissue infection; UTI, urinary tract infection; VABP, ventilator-associated bacterial pneumonia; VRE, vancomycin-resistant *Enterococcus.* Notes: ^a^ 4 g/d (post dialysis), 9 g/d (divided into 3 daily doses), 12 g/d (divided into 3 daily doses), 16 g/d (divided into 2 daily doses), 24 g/d (divided into 3 daily doses), [all were administered over a period of 15 min–1 h]; ^b^ 4 g LD + 8 g (divided into 4 daily doses) infused over 4 h, 4 g LD then 12 g (divided into 3 daily doses) infused over 6 h, 2 g LD then 6 g (divided into 3 daily doses) infused over 4 h, 4 g LD then 16 g (divided into 4 daily doses) infused over 4 h.

## Data Availability

The data used in the conduction of this study are available upon request.

## Supplementary Materials

The following supporting information can be downloaded at: https://www.mdpi.com/article/10.3390/antibiotics14121193/s1, Supplementary File S1: PRISMA checklist for the abstract; supplementary File S2: PRISMA checklist for the text; Supplementary File S3: Search strategies implemented in different resources. Ref. [107] is cited in Supplementary Materials.

## Abbreviations

BJI	bone and joint infection
CLSI	Clinical and Laboratory Standards Institute
CNS	central nervous system
CRAB	carbapenem-resistant Acinetobacter baumannii
ESBL	extended-spectrum β-lactamase
ESC	European Society of Cardiology
EUCAST	European Committee on Antimicrobial Susceptibility Testing
ICTRP	International Clinical Trials Registry Portal
KPC	Klebsiella pneumoniae carbapenemase
MBL	metallo-β-lactamase
MDR	multidrug resistant
MIC	minimal inhibitory concentration
MRSA	methicillin-resistant Staphylococcus aureus
MSSA	methicillin-susceptible Staphylococcus aureus
MurA	UDP-N-acetylglucosamine enolpyruvyl transferase
PD	pharmacodynamic
PDR	pandrug-resistant
PK	pharmacokinetic
PRISMA	Preferred Reporting Items for Systematic Reviews and Meta-Analyses
RCT	randomized controlled trial
RoB 2	Cochrane risk-of-bias tool for randomized trials
Robvis	risk-of-bias visualization
ROBINS-I V2	Risk Of Bias In Non-randomized Studies–of Interventions, Version 2
SOC	standard of care
SOFA	sequential organ failure assessment
UTI	urinary tract infection
VAP	ventilator-associated pneumonia
WHO	World Health Organization
XDR	extensively drug-resistant
