# Supplementary material for: Intravenous Fosfomycin for Gram-Negative and Gram-Positive Bacterial Infections: A Systematic Review of the Clinical Evidence
Source: Antibiotics (Basel). 2025 Nov 23;14(12):1193. doi: 10.3390/antibiotics14121193 (PMC12729521; doi:10.3390/antibiotics14121193)
Supplement: Supplementary file 1 [file antibiotics-14-01193-s001.zip › Supplementary File S3.pdf]

Supplementary file S3. Search strategies implemented in different resources (August 6, 2025, and August 12, 2025).

August 12, 2025

PubMed Central: 448 results

("bacterial infection" OR "bacterial infections" OR "gram-negative bacteria" OR "gram-positive bacteria" OR "gram-negative" OR "gram-positive") AND fosfomycin AND (monotherapy OR "combination therapy" OR "fosfomycin combination" OR "fosfomycin and") AND (therapy OR treatment OR cure) NOT "in vitro"

August 6, 2025

PubMed: 485 results

(("bacterial infections"[MeSH Terms] OR "gram-negative bacteria"[MeSH Terms] OR "gram-positive bacteria"[MeSH Terms] OR "bacterial infection"[tiab] OR "gram-negative"[tiab] OR "gram-positive"[tiab]) AND (fosfomycin[MeSH Terms] OR fosfomycin[tiab]) AND (monotherapy[tiab] OR combination therapy[MeSH Terms] OR "combination therapy"[tiab] OR fosfomycin combination[tiab] OR "fosfomycin and"[tiab]) AND (therapy[tiab] OR treatment[tiab] OR cure[tiab]))

Scopus: 350 results

(TITLE-ABS-KEY("bacterial infection" OR "gram-negative" OR "gram-positive") AND TITLE-ABS-KEY(fosfomycin) AND TITLE-ABS-KEY(monotherapy OR "combination therapy" OR "fosfomycin combination" OR "fosfomycin with") AND TITLE-ABS-KEY(therapy OR treatment OR cure))

Cochrane Library: 173 results

"bacterial infection" OR "gram-negative" OR "gram-positive" AND fosfomycin AND (monotherapy OR "combination therapy" OR "fosfomycin combination") AND (therapy OR treatment OR cure)

Web of Science: 119 results

("bacterial infection*" OR "gram-negative" OR "gram-positive") AND (fosfomycin) AND (monotherapy OR "combination therapy" OR "fosfomycin combination" OR "fosfomycin with") AND (therapy OR treatment OR cure)

Google Scholar: 7,850 results

"bacterial infection" OR "gram-negative" OR "gram-positive" fosfomycin (monotherapy OR "combination therapy" OR "fosfomycin combination") (therapy OR treatment OR cure)

Clinicaltrials.gov: 26 results

Bacterial infection AND fosfomycin

ICTRP: 1 result

fosfomycin AND (monotherapy OR combination therapy OR fosfomycin combination) AND (therapy OR treatment OR cure) AND (bacterial infection OR gram-negative OR gram-positive)
